# Astrocyte induction of disease-associated microglia is suppressed by acute exposure to fAD neurons in human iPSC triple cultures

**DOI:** 10.1016/j.celrep.2025.115777

**Published:** 2025-06-03

**Authors:** Alexandra M. Lish, Nancy Ashour, Richard V. Pearse II, Paige C. Galle, Gwendolyn A. Orme, Sarah E. Heuer, Courtney R. Benoit, Kellianne D. Alexander, Elyssa F.L. Grogan, Gizem Terzioglu, Allegra Scarpa, Andrew M. Stern, Nicholas Seyfried, Vilas Menon, Tracy L. Young-Pearse

**Affiliations:** 1 Ann Romney Center for Neurologic Diseases, Department of Neurology, Brigham and Women’s Hospital and Harvard Medical School, Boston, MA, USA; 2 Department of Biochemistry, Emory School of Medicine, Atlanta, GA, USA; 3 Center for Translational and Computational Neuroimmunology, Department of Neurology and the Taub Institute for the Study of Alzheimer’s Disease and the Aging Brain, Columbia University Irving Medical Center, New York, NY, USA; 4 Lead contact

## Abstract

Advancements in human induced pluripotent stem cell (hiPSC) technology have enabled co-culture models for disease modeling in physiologically relevant systems. However, co-culturing protocols face challenges in usability and consistency. Here, we introduce a robust, reproducible hiPSC-derived co-culture system integrating astrocytes, neurons, and microglia. This model leverages cryopreserved cells, enabling co-cultures within 20 days post-thaw. Comparing monocultures and tricultures, we demonstrate how cell-cell interactions shape transcriptional and functional states across all three cell types. Neurons in triculture exhibit increased spine density and activity, while astrocytes and microglia show altered responses to proinflammatory stimulation. Surprisingly, the presence of astrocytes induces upregulation of disease-associated microglia (DAM) genes, including *TREM2*, *SPP1*, *APOE*, and *GPNMB in microglia.* Additionally, while familial Alzheimer’s disease neurons induce a prototypical inflammatory response in microglia, the DAM signature is significantly dampened. Collectively, this study establishes a versatile human triculture model as a valuable resource for dissecting neuron-glia interactions and their role in neurodegenerative disease.

## In brief

Lish et al. present a co-culture system of iPSC-derived human microglia, astrocytes, and neurons to investigate intercellular communication across different environmental and genetic contexts. Transcriptomic and protein analyses show that co-culture drives distinct cell states, with astrocytes inducing disease-associated microglia. Notably, neurons with familial Alzheimer’s disease mutations disrupt this process.

## Graphical Abstract

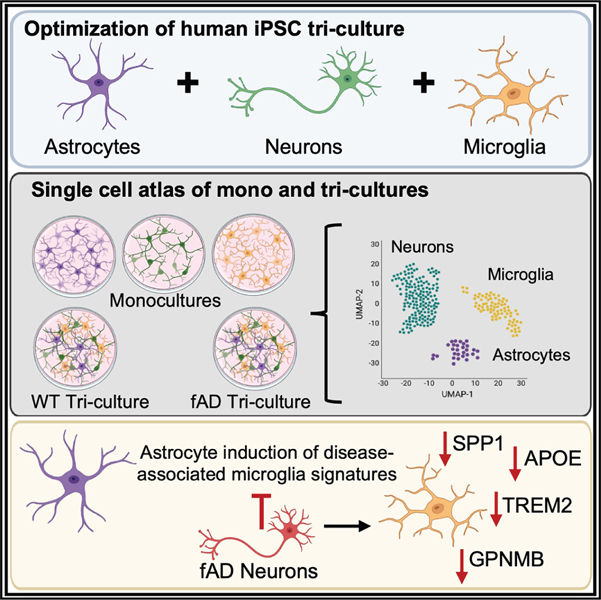

## INTRODUCTION

Inflammation is a hallmark of various neurodegenerative diseases, including Alzheimer’s disease (AD), and is prevalent in the aging brain (reviewed in Heneka et al.^1^). The primary immune responses in the brain are mediated in part by microglia and astrocytes, whose activation is intricately linked to neuropathological accumulation of aggregation-prone proteins such as amyloid-beta (Aβ) and tau (reviewed in Wu and Eisel^2^). Advances in single-cell RNA sequencing (scRNA-seq) have uncovered diverse microglial states that emerge in response to AD-related pathologies, including disease-associated microglia (DAMs).^3–11^ While DAMs are commonly associated with Aβ plaques in AD brains and mouse models during later disease stages, their temporal role, whether protective and/or pathogenic, is not yet clearly defined. Furthermore, the precise molecular and cellular mechanisms governing human microglial states remain incompletely understood. Postmortem analyses of human brain tissues have revealed intriguing hypotheses regarding how intercellular communication between glia and neurons regulates transcriptional states.^12,13^ However, a robust and reproducible human model system is required to effectively test these hypotheses.

Genetic studies further emphasize the role of glial cells in neurodegenerative diseases, with many AD risk genes expressed in microglia and astrocytes.^14,15^ *TREM2* and *APOE*, two strong genetic risk factors for late-onset AD (LOAD), have multifaceted roles in AD pathogenesis, including the modulation of inflammatory processes (reviewed in Martens et al.^16^ and Ulland and Colonna^17^). Furthermore, APOE is a ligand for TREM2,^18^ and both proteins are upregulated in DAMs.^4^ It has also been proposed that the TREM2-APOE pathway may act as a regulatory switch between homeostatic and activated microglia,^19^ and TREM2-knockout (KO) mice fail to switch from a homeostatic to a DAM state.^20,21^ However, the field’s understanding of APOE and TREM2 in microglial states largely relies on mouse models, which limits our ability to discern species-specific differences and unravel the mechanisms of intercellular crosstalk that regulate human microglial activity.

Human induced pluripotent stem cell (hiPSC) technology provides a valuable complement to mouse models for studying disease pathogenesis by incorporating a human genomic context into a highly controlled and manipulable environment (reviewed in Young-Pearse et al.^22^). Advances in iPSC technology have introduced a variety of cellular models, including co-cultures of neurons, astrocytes, and microglia.^23–28^ These models more accurately mimic the cellular interactions of the human brain, yet they come with significant challenges. Co-cultures often utilize differentiation protocols that require extended maturation periods exceeding 90 days, and achieving consistent, reproducible ratios of cell-type proportions remains a significant challenge.^24,26,29^ Automated culturing platforms have emerged as a promising solution, enabling more consistent and controlled cell fates of iPSC-derived cells.^30^ However, the technical complexity and cost of these platforms have hindered their wide-spread adoption, limiting the establishment of standardized co-culture protocols. Further development of user-friendly, scalable, and consistent systems will be crucial to making these advanced models accessible across the broader research community.

Here, we present the development of a robust and facile hiPSC-derived triculture (TC) model consisting of iPSC-derived astrocytes (iAs), neurons (iNs), and microglia (iMGs). Initially, each cell type is independently differentiated: iNs and iAs are generated using virally introduced NGN2 and SOX9/NFIB transcription factors, respectively, and microglia are differentiated using a hematopoietic precursor intermediate.^31–33^ After differentiation, iAs and iMGs are re-plated onto iNs, followed by co-culture. The incorporation of cryopreservation steps during differentiation enables co-cultures to be initiated within 20 days of thaw and allows for quality control assessments of each cell type prior to the initiation of co-culture. Using this system, we perform a detailed characterization of each cell type in monoculture (MC) and TC, revealing how intercellular interactions shape transcriptional states and functional properties across all three cell types. scRNA-seq analysis highlights key transcriptional differences between mono- and co-cultures, including upregulation of pathways related to synaptic organization and assembly in neurons, reflected by an increase in dendritic spine density, synaptic vesicle (SV) release, and activity. Astrocytes exhibit upregulation of cholesterol efflux and cell adhesion pathways in TC, while microglia show increased expression of genes related to lipoprotein catabolism and microtubule cytoskeleton organization, alongside a more ramified morphology. Furthermore, the co-culture environment replicates transcriptional states characterized in human postmortem brain tissue, including the emergence of a human DAM signature in a subset of microglia. Notably, TREM2, APOE, GPNMB, and SPP1 protein levels are significantly and strongly upregulated in TC, a process driven by astrocytes. Additionally, we demonstrate the utility of our model by investigating glial responses to neurons carrying familial AD (fAD) mutations. While microglia co-cultured with fAD neurons exhibit some proinflammatory characteristics, the DAM-associated gene signature is notably diminished. Remarkably, the presence of high Aβ-secreting fAD neurons suppresses astrocyte-dependent upregulation of APOE, TREM2, SPP1, and GPNMB in microglia, highlighting a key disruption in neuron-glial cell communication. Collectively, our findings establish this triculture model as a valuable resource for studying neuron-astrocyte-microglial cell interactions in a human context, with applications for investigating neurodegenerative disease mechanisms. As a recent example, this model revealed a critical role for microglia in mediating astrocyte CLU-dependent synapse loss and tau phosphorylation in neurons.^34^

## RESULTS

### Development of an iA-iN-iMG TC platform

To optimize a protocol for TC of iAs, iNs, and iMGs, we employed iPSC-derived cell lines from two donors who were part of the Religious Orders and Rush Memory and Aging Project (ROSMAP): one male (BR33) and one female (BR24).^35^ Both individuals were over 85 years old at the time of death, were not cognitively impaired, and had no neurological diagnoses. In subsequent sections, we also utilize a previously described neuronal line carrying homozygous fAD mutations in amyloid precursor protein and presenilin 1 (APP^Swe^; PSEN1^M146V^) and its wild-type (WT) isogenic control line (APP; PSEN1 WT).^36,37^ The lines used in each subsequent figure are detailed in Table S1.

**Figure 1.**
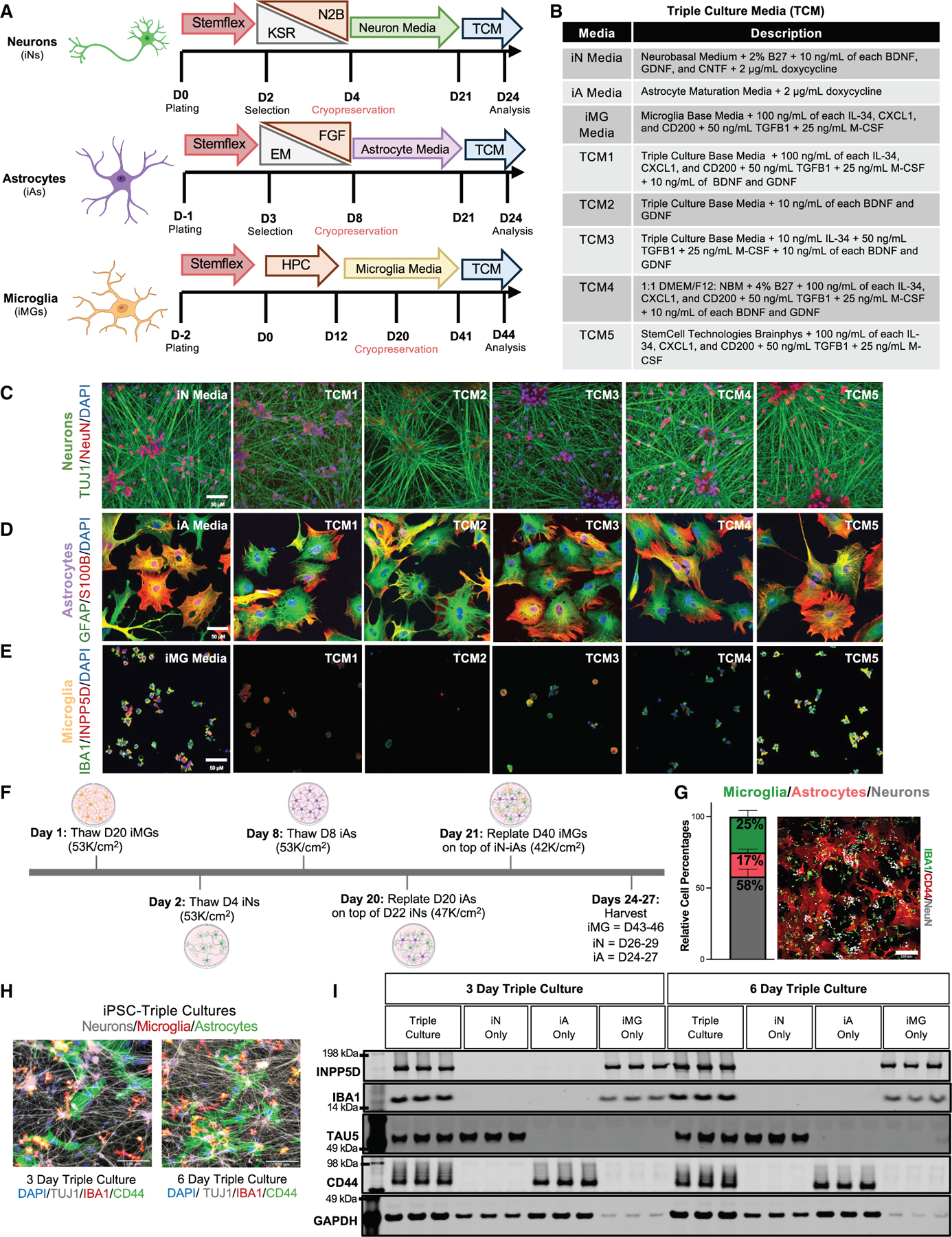
Selection of the optimal media for triculture of human iPSC-derived neurons, astrocytes, and microglia (A) Schematic of the iPSC differentiation protocols for neurons (iNs), astrocytes (iAs), and microglia (iMGs). iNs and iAs were generated by lentiviral expression of lineage-specific transcription factors, while iMGs were derived via a hematopoietic precursor (HPC) stage using non-viral methods. Cryopreservation days are indicated; each cell type was fully differentiated before switching to triculture media (TCM). Key abbreviations: KSR, knockout serum replacement; N2B, neurobasal with N2/B27; EM, expansion medium; FGF, fibroblast growth factor medium. (B) Table summarizing composition of each cell-type-specific media and TCM (see Table S2 for full details). (C–E) Representative immunostaining of neurons (C), astrocytes (D), and microglia (E) maintained in either their respective media or TCM. Markers shown include TUJ1/NeuN for neurons, GFAP/S100B for astrocytes, and IBA1/INPP5D for microglia. Scale bars, 50 μm. (F) Timeline of the triculture workflow. Cryopreserved iNs, iAs, and iMGs were thawed and matured separately; astrocytes and microglia were sequentially plated onto neuron cultures on days 20 and 21 and then co-cultured for 3–6 days. The days in bold refer to the start of thawing the first stock of cryopreserved cells, and the non-bolded days refer to the day of differentiation for each individual cell type. (G) Bar plot showing the relative percentages of NeuN^+^ (neurons), IBA1^+^ (microglia), and CD44^+^ (astrocytes) cells at day 27, determined from immunostaining (*n* = 2 genetic backgrounds, 3 differentiations, and 2 wells per differentiation). Error bars represent standard error. See Figure S1E for individual well values. A representative field of view (FOV) is shown, with six FOVs per well analyzed by blinded quantification. Scale bar, 200 μm. (H) Representative triculture images (3–6 days of co-culture) labeled for CD44 (astrocytes), IBA1 (microglia), and TUJ1 (neurons). Scale bars, 100 μm. (I) Western blot of tricultures at days 3 and 6, probed for INPP5D, IBA1, TAU5, CD44, and GAPDH, confirming the presence of all three cell types.

We first evaluated five TC media (TCM) formulations using MCs of each cell type (Figures 1A and 1B; Table S2). Three of the five TCMs were based on a TC base medium, which included supplements and growth factors standard in iA and iMG MC media (see STAR Methods). TCM4 used a 1:1 mixture of neurobasal media (NBM) and DMEM/F12 as the base media, reflecting the media composition used in iN, iMG, and iA cultures. TCM5 was composed of StemCell Technologies BrainPhys Neuronal Culture Media and SM1 kit (catalog #05792). Each MC (iNs, iMGs, and iAs) was fully differentiated as previously described.^31–33,35,38–40^ After differentiation, cells were switched to one of five TCM conditions (TCM1–5) or maintained in their respective MC media. In certain TCMs, we omitted some growth factors and cytokine supplements that are typically included in MC media. Since each cell type was fully differentiated before co-culture, the presence of other cell types may eliminate the need for these factors, and some factors that promote the health and differentiation of one cell type could have detrimental effects on others. After 3 days in TCM, the cells were either fixed or lysed to assess cell-type markers via immunostaining and western blot (WB) (Figures 1C–1E and S1A–S1C). Astrocyte and neuron markers showed relatively consistent patterns across the different media conditions, whereas microglia appeared more sensitive to changes in media composition. Notably, iMGs did not survive in TCM2, which lacked the growth factors and cytokines used in standard iMG differentiation. Furthermore, TCM3, which included only three of the five iMG differentiation cytokines, resulted in compromised microglial morphology and marker expression. It’s important to note that astrocytes are quite heterogeneous, displaying multiple morphologies within the same well (Figure S1D). While the majority of astrocytes in maturation media exhibited morphologies similar to those shown in Figure 1D, a smaller subset displayed more extensive branching (Figure S1D), and this population was reduced in all other media conditions tested. Based on these MC results, we selected TCM1, TCM4, and TCM5 for further analysis in TCs. TCM4 and TCM5 were chosen for their favorable immunostaining morphology and cell-type marker expression. Although microglial morphology appeared more rounded in TCM1, this media was included for TC testing because it contained all of the growth factors typically used for iN, iA, and iMG differentiation.

Although analyzing morphology and cell-type-specific marker expression in MCs is informative, it is critical to evaluate media conditions in TCs as each cell type secretes factors that promote and maintain the identity of other cell types. To establish TCs, each cell type was initially plated and differentiated separately as outlined in Figure 1F. Unless otherwise noted, the days of differentiation (day 0, day 20, etc.) referred to subsequently are based on the TC timeline outlined in Figure 1F, which begins with the thawing of the first set of cryopreserved cells. On day 2, iNs are thawed and plated in their final destination, and on day 20, iAs are dissociated and re-plated on iNs. On day 21, iMGs are dissociated and added to the iN-iA co-cultures. TCs were maintained in TCM for an additional 3–6 days. Cells were initially seeded at a ratio of approximately 10:9:8 (iNs:iAs: iMGs). By the end of the co-culture period (day 27), the cultures consisted of an approximate ratio of 6:2:3 (iNs:iAs:iMGs) (Figures 1G and S1E), likely reflecting differences in cell adhesion and proliferation across cell types (see STAR Methods for more details). By TC day 27, iNs had been differentiated for 29 days, iAs for 27 days, and iMGs for 46 days. To determine the optimal media for TC, we compared TCM1, TCM4, and TCM5 across paired MCs and TCs (Figures S1F and S1G). All TCs expressed cell-type-specific markers for each tested media condition. In TCM1, we observed a reduced expression of microglia markers and a more amoeboid morphology (Figures S1F and S1G). In contrast, iMGs in TCM4 and TCM5 displayed a more ramified morphology, characterized by long processes and highly arborized branches. Given that TCM1 contains eight supplements and growth factors from iA maturation media (see STAR Methods), we introduced each component separately to iMG MCs. The addition of dibutyryl cAMP (dbcAMP) at 500 μg/mL led to significant cell death (data not shown). Based on this observation, we established TCM6, which consists of a 1:1 ratio of BrainPhys to DMEM/F12/NBM and includes all iA supplements and growth factors except dbcAMP (Figure S1H; Table S2). We compared TCM6 to TCM5 to determine whether iA morphology differs signficantly in the presence of additional astrocyte growth factors and supplements. However, both media conditions yielded similar results, with robust expression of cell-specific markers and characteristic star-like morphology of astrocytes (Figures S1I and S1J). While TCM4–6 each exhibited high expression of cell-type markers as demonstrated by WB and immunostaining, we chose to proceed with TCM5 for future experiments, including paired analyses of MCs and TCs. TCM5 contains BrainPhys, a medium optimized for maintaining iN differentiation and that has been used in other co-culture models.^30,31,41^ Comparison of 3- and 6-day TCs showed consistent and robust expression and morphology of cell-type markers (Figures 1H and 1I), confirming the stability of these cultures. All subsequent results employ a 6-day co-culture of the cell types under study unless otherwise noted.

### Co-culture increases neuronal spine density and activity and reduces glial inflammatory responses

**Figure 2.**
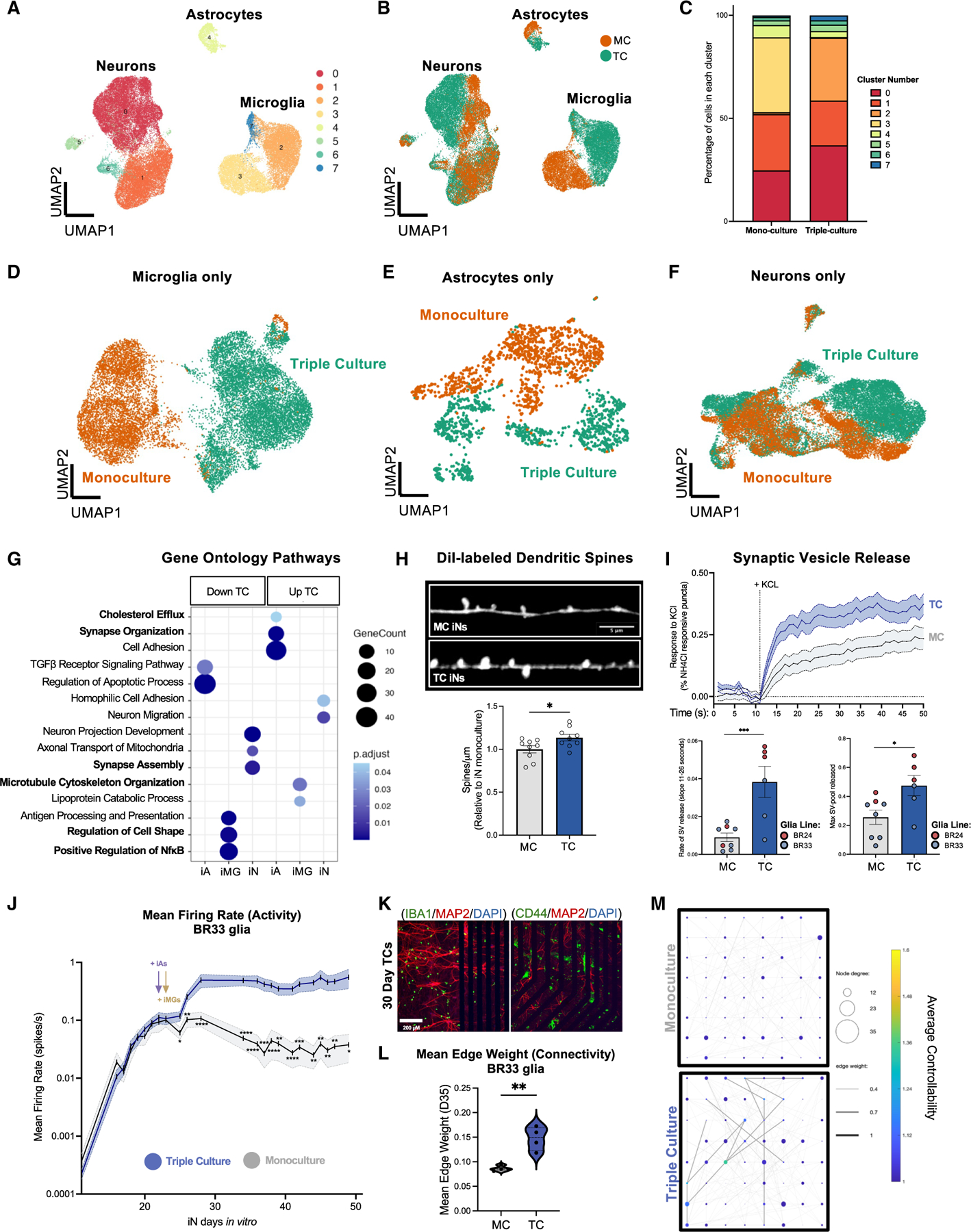
Triculture enhances neuronal spine density and mean firing rate (A and B) scRNA-seq was performed on monocultures (MCs) of each cell type and on tricultures (TCs) after 6 days of co-culture (total 58,322 cells). UMAP plots show clustering by marker expression, with clusters labeled by cluster index in (A) and by cell/culture type in (B). For all scRNA-seq data, one genetic background was used. See Figures S2A–S2G for differentiation markers. (C) Proportional composition of each cluster, color coded to match (A). (D–F) Microglial cell, astrocyte, and neuron populations were isolated, re-clustered, and colored by culture condition (MC, orange; TC, green). (G) Dot plot of significantly enriched Gene Ontology (GO) pathways in iNs, iAs, and iMGs (DEGs from Figure S2H). Dot size reflects the number of DEGs in each pathway; color indicates adjusted *p* value. See Table S3 for additional details. (H) DiI(1,1’-dioctadecyl-3,3,3’,3’-tetramethylindocarbocyanine perchlorate) labeling of neuronal dendritic spines in MC and TC. Spine density was measured by reconstructing dendritic cables and counting spines per dendrite length; two genetic backgrounds for glial cells; *n* = 3 independent co-cultures and 3 wells per co-culture, with blinded analysis. Each dot represents the average of five dendrites per well; *p < 0.05 by mixed-effects model. Scale bar, 5 μm. (I) Synaptic vesicle (SV) release was assessed in MC and TCs using live-cell imaging of SypHy, a pH-sensitive fusion protein that fluoresces upon vesicle fusion and exposure to extracellular pH (7.4) after KCl stimulation. iNs were transduced with SypHy-expressing lentivirus on day 19, with iAs added on day 20 and iMGs on day 21. On day 27, iNs were imaged before and during KCl stimulation, followed by NH_4_Cl treatment to unquench SypHy fluorescence and identify responsive puncta (≥2-fold fluorescence increase). Shown are representative traces from BR33 MCs and TCs depicting the average SypHy signal (±SEM) of all NH_4_Cl-responsive puncta over time. Quantifications show the rate of SV release immediately after KCl stimulation (11–26 s) and the maximum SV pool released. *N* = 2 genetic backgrounds, 3 independent co-cultures, and 1–3 wells per co-culture. ***p < 0.001 and *p < 0.05, mixed-effects model. (J) Mean firing rate (spikes/s) of neurons from day 11 to day 49 in MC versus TC, recorded on Axion multielectrode arrays (MEAs) (64 electrodes/well, 4 wells/condition, and 1 differentiation). BR33 glia cells were used; see Figure S3A for BR24 glia. Mixed-effects analysis with Sidak’s multiple comparisons, ****p < 0.0001, ***p < 0.001, **p < 0.01, and *p < 0.05. (K) Representative immunostaining of MEA wells at iN day 50 (after 28 days of co-culture) showing CD44 (astrocytes), IBA1 (microglia), MAP2 (neurons), and DAPI (nuclei). Scale bar, 200 μm. (L) Mean edge weight (a measure of connectivity strength) on iN day 35 comparing TC to MC. BR33 glia cells were used; see Figure S3B for BR24 glia. *N* = 64 electrodes/well, 4 wells/condition, and 1 differentiation. Dots correspond to each individual well. Unpaired Student’s t test, **p < 0.01. (M) Representative network graphs on iN day 35 illustrating microscale network organization in MC and TC. Node strength (circle size) represents the influence of individual electrodes, edge weight (line thickness) indicates connection strength, and node color reflects average controllability, which quantifies the ability of individual nodes to facilitate transitions between different network states.

For all subsequent comparisons between MCs and TCs, samples were cultured in parallel under the exact same media and plating conditions. To characterize cells in MC versus TC, we first performed an initial scRNA-seq analysis in one genetic background. All key findings from this analysis were then independently replicated in at least three differentiations and two genetic backgrounds (BR24 and BR33). Uniform manifold approximation and projection (UMAP) dimensional reduction revealed three major clusters corresponding to neurons, astrocytes, and microglia, with a total of eight subclusters (Figure 2A). Cells in each culture condition showed minimal expression of pluripotency markers and an enrichment of excitatory neuron markers^42^ for iNs, astrocyte markers^43^ for iAs, and microglial markers^11^ for iMGs (Figures S2A–S2G). Although astrocytes were estimated to comprise ∼17% of the cultures based upon immunostaining (Figure 1G), their recovery for scRNA-seq was poor, reflecting similar findings in human brain studies where astrocytes account for only 2%–9% of total nuclei recovered.^44^ This underrepresentation may be due to the preferential loss of astrocytes during preparation, likely because of their distinct biochemical and biophysical properties.^45^ Additionally, neurons express up to 10 times more RNA than astrocytes, contributing to their overrepresentation in the final library composition.^46^ Regardless, sufficient numbers of cell fates were analyzed to observe significant shifts in cluster identities between MCs and TCs within each cell type (Figures 2B and 2C). Independent clustering analyses for each cell type revealed that, while neurons shared some clusters between TC and MC, astrocytes and microglia in TC formed distinct clusters compared to those in MC (Figures 2D–2F). Previously, we characterized iAs using scRNA-seq and identified three major clusters.^47^ Marker genes from clusters identified in our previous analysis also labeled distinct clusters of iAs in this dataset (Figure S2F).

Next, we performed differential expression analysis between MCs and TCs (Figure S2H; Table S3) to identify cell-type-specific responses to co-culture. Gene Ontology (GO) analyses revealed distinct pathway enrichments for each cell type when present in TC (Figure 2G; Table S3). Notably, pathways involved in synapse organization, synapse assembly, and cell adhesion were significantly altered in TC. Additionally, GO cellular component analysis of neurons in TC indicated that many differentially expressed genes (DEGs) encoded proteins that are localized to dendrites (Table S3). Consequently, we measured dendritic spine density in paired neuron MCs and TCs and observed a significant increase in spine density in neurons cultured with astrocytes and microglia (Figure 2H). Given these transcriptional and structural differences, we next assessed synaptic vesicle (SV) release in MCs and TCs (Figure 2I) using live-cell imaging of SypHy as previously described.^48^ SypHy is a pH-sensitive protein that fluoresces when SVs fuse and are exposed to extracellular pH. SV release was triggered by potassium chloride (KCl) stimulation, and fluorescence changes were measured over time to assess release activity. Both the maximum rate of SV release and the initial rate of release following KCl stimulation were significantly increased in TCs compared to MCs (Figure 2I). The steeper slope of release may reflect a greater pool of vesicles docked and primed for release, indicating that glial cells may influence both SV availability and release dynamics. These findings, along with the observed increase in dendritic spine density, support a role for glial cells in promoting synaptic function. Building on this observation, we used multielectrode array (MEA) recordings to compare mean firing rate and network dynamics in MCs and TCs (Figures 2J–2M and S3A–S3C). Recordings started on iN day 11—10 days before glia were added—and continued through iN day 49 (BR33 glia) or day 35 (BR24 glia). Neuronal firing (mean firing rate) was consistently higher in TCs compared to MCs across two glial genetic backgrounds (Figures 2J and S3A), with this difference emerging only after glial introduction and persisting thereafter. Immunostaining of MEA wells confirmed the sustained presence of all three cell types after 30 days in TC (Figure 2K). To assess network connectivity, we employed the MATLAB-based MEA Network Analysis Pipeline (MEA-NAP),^49^ revealing that mean edge weight—a measure of electrode-to-electrode connection strength—was also elevated in TCs (Figures 2L–2M, S3B, and S3C). Overall, these data demonstrate that glial cells enhance both neuronal activity and connectivity in this co-culture system.

**Figure 3.**
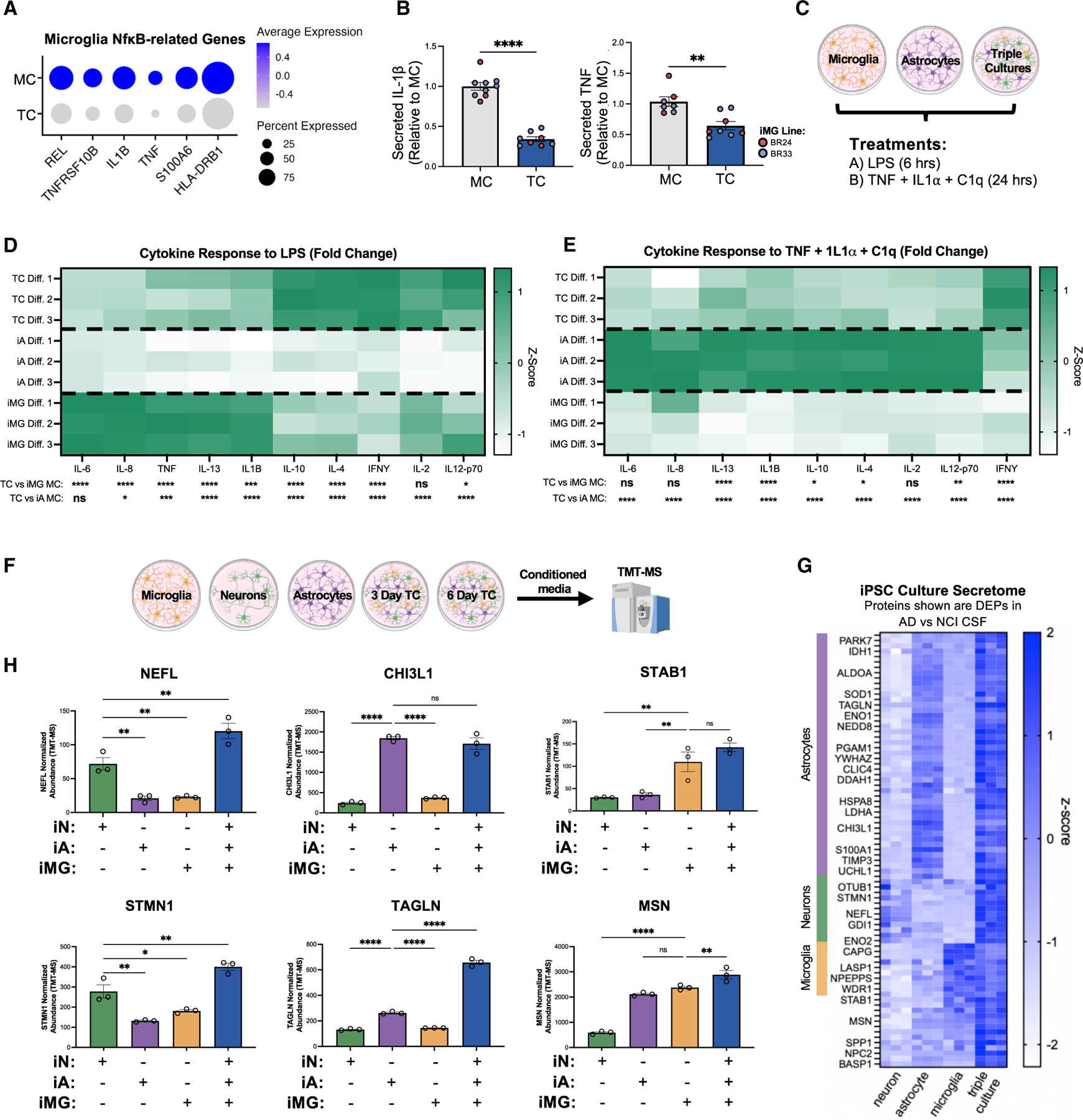
The co-culture environment influences glial responses to inflammatory stimuli (A) Dot plot of leading-edge DEGs (upregulated in iMGs from TC versus MC related to NF-κB signaling; see Figure 2G). Dot size indicates the fraction of cells expressing each gene; color indicates adjusted *p* value, with all genes shown having a *p*-adj. < 0.05. (B) Secreted levels of TNF and IL-1β in paired MCs and TCs were measured via ELISA. Since cells in MCs and TCs were plated in parallel at equal densities, the MC values represent the total cytokine levels detected in both iA and iMG MCs. *N* = 2 genetic backgrounds, 3 differentiations, and 2–3 wells/differentiation. Mixed-effects model. See Figures S3D and S3E for the measurements separated by iA and iMG MCs. (C) Schematic of culture conditions and inflammatory treatments (LPS or TNF + IL-1α + C1q). Cytokines were measured in the media using the MesoScale V-Plex panel; iN MC values were not in detection range, as expected, and data are not shown. (D and E) Heatmaps of cytokine responses (fold change) in iA/iMG MCs and TCs treated with LPS for 6 h (D) or TNF + IL-1α + C1q for 24 h (E). Data are shown as *Z* scores across culture conditions; *N* = 2 genetic backgrounds, 3 differentiations, and 3 wells/differentiation. Technical replicates were averaged for each differentiation. See Table S4 for full data. Two-way ANOVA with Dunnett’s multiple comparisons test. Diff, differentiation. (F) Schematic of secretome profiling. Conditioned media from MCs and TCs were collected in parallel and analyzed by tandem mass-tag (TMT) mass spectrometry (1 genetic background, 3 independent wells per condition). See also Table S5. (G) Heatmap of selected proteins identified in secretome profiling that are differentially expressed in Alzheimer’s disease (AD) versus not cognitively impaired (NCI) cerebral spinal fluid (CSF).^53^ (H) Expression of six AD-related proteins (NEFL, STMN1, CHI3L1, TAGLN, STAB1, and MSN) that are highly expressed in TCs. One-way ANOVA with Sidak’s multiple comparisons. Data are from the secretome experiment described in (F). Data are the mean ± SEM. ****p < 0.0001, ***p < 0.001, **p < 0.01, *p < 0.05, and ns, not significant.

Pathway analyses also identified genes downregulated in TC iMGs that are relevant to the NF-κB pathway (Figures 2G and 3A; Table S3). To validate this observation, we measured levels of interleukin-1β (IL-1β) and tumor necrosis factor (TNF) in the media from paired glial MCs and TCs and found that the secreted levels of these cytokines were reduced in TCs (Figures 3B, S3D, and S3E). We next sought to explore how cells in MCs and TCs respond to inflammatory stimuli by exposing cells in MCs and TCs to two inflammatory paradigms: lipopolysaccharide (LPS) to activate TLR4 signaling in microglia^50^ and TNF + IL-1α + C1q to induce astrocyte reactivity^47,51,52^ (Figure 3C). We assessed the response by measuring the fold changes in a panel of 10 cytokines following stimulation (Figures 3D and 3E; Table S4). As expected, LPS stimulation predominantly affected iMG MCs and TCs. Notably, the fold increase of anti-inflammatory cytokines such as IL-4 and IL-10 was significantly higher in TCs than in iMG MC, while proinflammatory cytokines such as IL-6 and TNF were significantly lower (Figure 3D; Table S4). While the TNF + IL-1α + C1q cytokine cocktail triggered responses in both iA and iMG MCs, the response was much greater in iA MCs (Figure 3E; Table S4). Moreover, the cytokine response in TCs was significantly attenuated compared to iA MC, with this trend observed for both pro- and anti-inflammatory cytokines, except for interferon-γ (IFN-γ), which was elevated in TCs.

We next analyzed the secretome profiles of iNs, iAs, and iMGs in both MCs and TCs in an unbiased manner using tandem mass-tag mass spectrometry (TMT-MS) of conditioned media (Figure 3F; Table S5). Identifying secreted proteins is important, as it enables (1) investigation of factors involved in intercellular signaling between cell types and (2) correlation of the secretome with plasma and cerebrospinal fluid (CSF) biomarker data from human studies. Our analysis revealed 515 unique proteins, many of which are proteins detected in CSF that are differentially expressed in AD (Figure 3G).^53^ Notably, many of these biomarkers, including NEFL, CHI3L1, and MSN, showed higher expression levels in the TC secretome compared to MC, underscoring the value of comparing both culture types for investigating key proteins of interest (Figure 3H).

**Figure 4.**
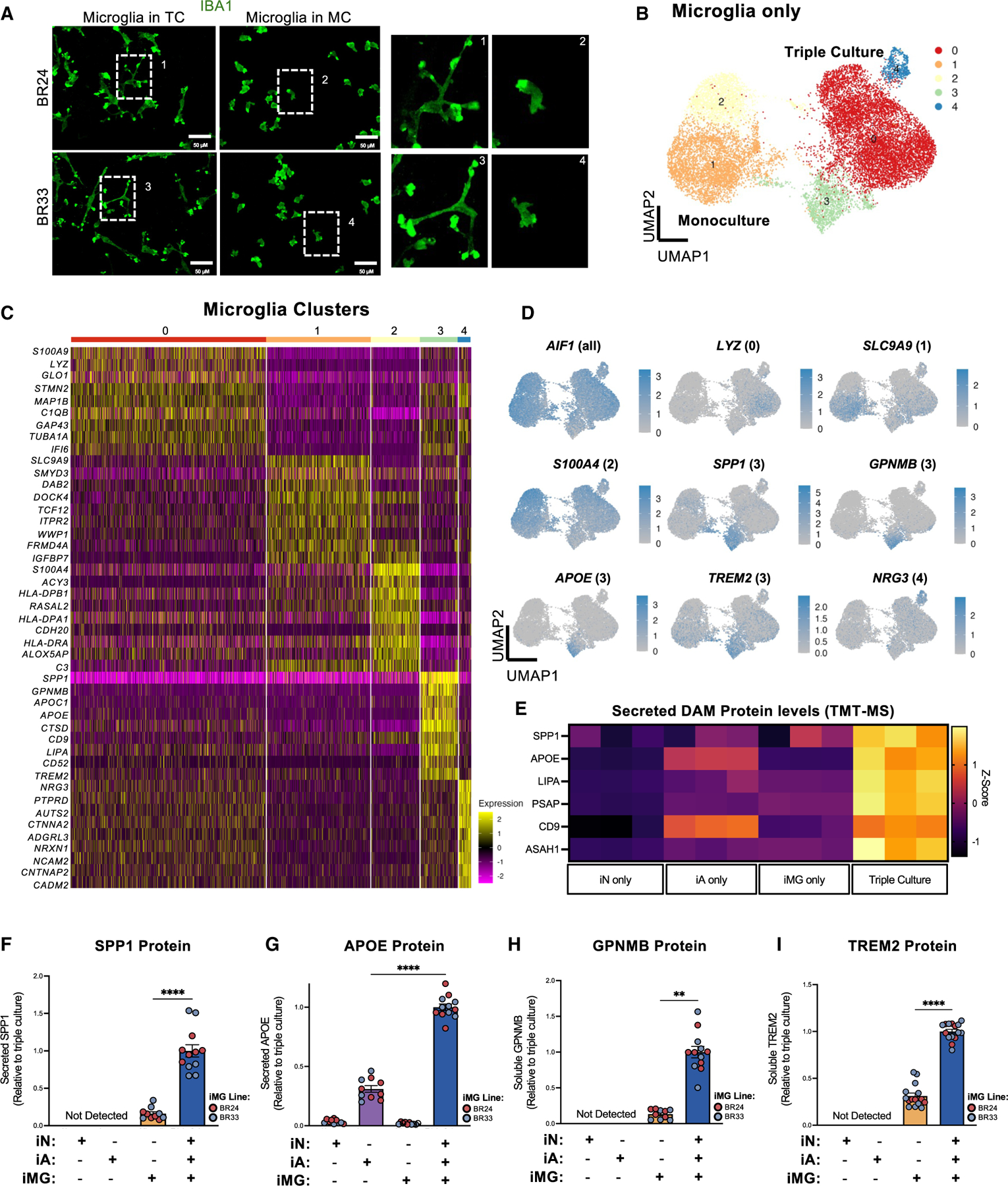
TC induces diverse transcriptional states in microglia (A) Representative images of microglia (green, IBA1) in MC versus TC for two genetic backgrounds (BR24 and BR33). Scale bars, 50 μm. Insets show higher-magnification views; see Figure S3F for additional images. (B) UMAP plot of microglia, subclustered and labeled by cluster identity. Microglial data are subsetted from the scRNA-seq experiment shown in Figure 2; see Figures S3G and S3H for neuron and astrocyte subclusters, respectively. (C) Heatmap of DEGs across microglial cell clusters, organized by hierarchical similarity and microglial state. See Figures S3I and S3J for neuron and astrocyte marker data, respectively. See also Table S3. (D) UMAP feature expression plots of nine selected microglial cell cluster markers: *AIF1* (general microglia), *LYZ* (cluster 0), *SLC9A9* (1), *S100A4* (2), *SPP1/GPNMB/APOE/TREM2* (3), and *NRG3* (4). See Figure S2I for cluster 3 marker expression in all cell types. (E) TMT-MS-based secretome profiling from paired MC/TC microglia (from experiment depicted in Figure 3F). Heatmap shows secreted levels of disease-associated microglia (DAM)-related proteins (SPP1, APOE, LIPA, PSAP, CD9, and ASAH1). The full dataset is in Table S5. See Figure S4A for bar graphs of each protein. (F–I) ELISA quantification of SPP1, APOE, GPNMB, and TREM2 in media from paired MCs and TCs, normalized to TC values within each experiment (*N* = 2 genetic backgrounds, 4–5 differentiations, and 2–3 wells/differentiation). Data are the mean ± SEM. Statistical comparisons used a mixed-effects model; ****p < 0.0001 and **p < 0.01.

These findings collectively indicate that the co-culture environment significantly influences glial inflammatory responses and secretory profiles. Supporting this interpretation, our scRNA-seq analysis revealed that iMGs in TC showed differential expression of pathways related to cell shape and microtubule cytoskeletal organization (Figure 2G; Table S3). Since microglial morphology is highly dynamic in response to environmental stimuli and reflects their functional state (outlined in Paolicelli et al.^9^), these transcriptomic changes align with alterations in inflammatory signaling and correspond to the pronounced morphological differences observed between paired iMG MCs and TCs (Figures 4A and S3F).

### Astrocytes induce human DAM signatures in microglia

To further investigate cell-type-specific responses to the co-culture environment, we isolated and re-clustered our scRNA-seq data based on primary cell type (i.e., microglia, astrocytes, and neurons). We then performed differential gene expression analysis to compare MC and TC within microglial cell clusters (Figures 4B and 4C; Table S3), within neuron clusters (Figures S3G and S3I; Table S3), and within astrocyte clusters (Figures S3H and S3J; Table S3). First focusing on the microglial subclusters, we leveraged previously published data and identified iMG states similar to those identified in postmortem human brain tissue.^11,54,55^ These included an antigen-presenting state (cluster 2, expressing *HLA-DPA1*, *HLA-DPB1*, and *HLA-DRA*), a DAM-like state (cluster 3, expressing *APOE*, *TREM2*, *GPNMB*, *SPP1*, *CD9*, and *LGALS3*), and a synapse-associated state (cluster 4, expressing *NRG3*, *CNTNAP2*, and *NCAM2*) (Figures 4C and 4D). Notably, *MITF* was also upregulated in cluster 3 (Table S3) and has been recently described as a key transcription factor that regulates DAM signatures.^11^ Interestingly, within astrocytes, a disease-associated-like cluster was also identified (cluster 3, expressing *SPP1*, *FTL*, *ALOX5AP*, *GPNMB*, *MAFB*, *HLA-DRA*, and *CYBB*) as well as a synapse-associated state (cluster 1, expressing *ROBO1*, *PTPRD*, *NRG3*, and *NRXN1*) (Figure S3J; Table S3).

**Figure 5.**
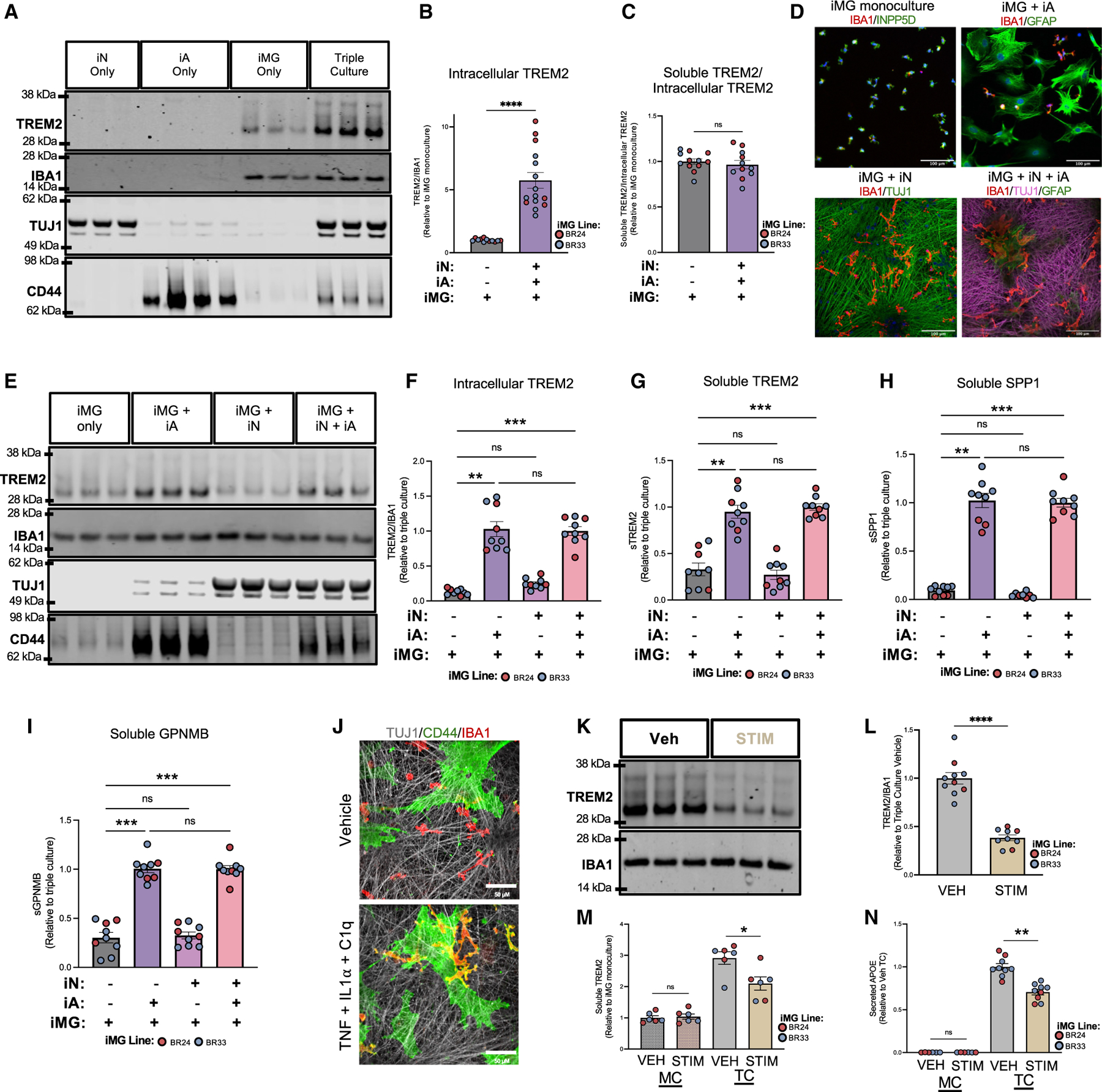
DAM signature upregulation in TC is dependent on astrocyte-microglial intercellular communication (A) Representative western blot (WB) from one genetic background (technical replicates from a single differentiation) showing TREM2, IBA1, TUJ1, and CD44 across MC and TC. (B) WB quantification of TREM2 (normalized to IBA1) in microglial MC versus TC, expressed relative to microglial MC. *N* = 2 genetic backgrounds, 5 differentiations, and 2–3 wells/differentiation. (C) Ratio of soluble TREM2 (ELISA) to intracellular TREM2 (WB) in the same wells, normalized to microglial MC. *N* = 2 genetic backgrounds, 5 differentiations, and 2–3 wells/differentiation. (D) Immunofluorescence illustrating iMG-alone cultures (IBA1^+^, INPP5D^+^), iMG-iA co-cultures (IBA1^+^, GFAP^+^), iMG-iN co-cultures (IBA1^+^, TUJ1^+^), and iMG-iN-iA TCs (IBA1^+^, TUJ1^+^, GFAP^+^). Scale bars, 100 μm. (E) Representative WB (one genetic background, technical replicates from a single differentiation) probing TREM2, IBA1, TUJ1, and CD44 in the same co-culture conditions as in (D). (F–I) Quantification of intracellular TREM2 (WB) and soluble (ELISA) TREM2, SPP1, and GPNMB, normalized to TC within each experiment. *N* = 2 genetic backgrounds, 3 differentiations, and 3 wells/differentiation. (J) TCs treated with vehicle or TNF + IL-1α + C1q for 24 h, stained for neurons (TUJ1, gray), astrocytes (CD44, green), and microglia (IBA1, red). Scale bars, 50 μm. (K and L) Representative WB (one genetic background, technical replicates from a single differentiation) of TREM2 and IBA1 in TCs ± TNF + IL-1α + C1q, with quantification (TREM2/IBA1) normalized to vehicle controls. *N* = 2 genetic backgrounds, 3 differentiations, and 3–4 wells/differentiation. (M and N) Soluble TREM2 and secreted APOE (ELISA) in iMG MCs and TCs treated with vehicle or TNF + IL-1α + C1q. *N* = 2 genetic backgrounds, 2–3 differentiations, and 3 wells/differentiation. See Figures S4F and S4G for SPP1 and GPNMB. Data are the mean ± SEM; statistics by mixed-effects model. ****p < 0.0001, ***p < 0.001, **p < 0.01, *p < 0.05, and ns, not significant.

Although we observed that NF-κB-dependent inflammatory responses were downregulated in TC iMGs (Figures 2G and 3A), the unexpected discovery of a DAM-like state in microglia in TC underscores the complex role of neurons and astrocytes in modulating microglial immune activity. To determine whether the presence of this DAM cluster reflected an upregulation of these genes at the protein level in TCs, we revisited our secretome profiling data (Table S5). Six of the top 20 DAM markers defining cluster 3 at the RNA level (Table S3) were detected in the secretome dataset, and among these, SPP1, APOE, LIPA, ASAH1, and PSAP were all upregulated at the protein level in TCs (Figures 4E and S4A). We validated the secreted levels of APOE and SPP1 by ELISA, observing a pronounced and significant increase in TCs compared to MCs (Figures 4F and 4G). However, since APOE is more highly expressed in astrocytes and both SPP1 and APOE were also upregulated in iAs in TCs (Figures S2I, S4B, and S4C), it is difficult to conclusively determine the proportion of microglial cell- versus astrocyte-secreted levels in the TC. Therefore, we measured the levels of TREM2 and GPNMB, two DAM markers more restricted to microglial expression (Figures S2I, S4D, and S4E). Both TREM2 and GPNMB are type I transmembrane proteins that can be cleaved by proteases such as ADAM10, resulting in soluble forms that are detectable in the conditioned media of cell cultures. Similar to APOE and SPP1, soluble levels of GPNMB and TREM2 were significantly upregulated in TCs (Figures 4H and 4I). Intracellular TREM2 protein also was markedly upregulated in TC, and the ratio of soluble to intracellular TREM2 was unchanged (Figures 5A–5C). This suggests that the rise in soluble TREM2 levels in TC reflects an overall increase in protein expression rather than enhanced cleavage activity.

Next, we investigated whether astrocytes or neurons alone could induce DAM upregulation by establishing the following culturing conditions: iMG MC, iMG-iA co-culture, iMG-iN co-culture, and iMG-iA-iN TC (Figure 5D). Although co-culture with iNs dramatically altered microglial morphology, only the presence of astrocytes upregulated TREM2, SPP1, and GPNMB protein expression (Figures 5E–5I). Given the dynamic nature of TREM2 *in vivo* and its response to inflammatory signals,^56,57^ we determined whether enhancing astrocyte reactivity could affect TREM2 expression. Indeed, treating TCs with a reactive astrocyte-inducing cocktail of TNF + IL-1α + C1q significantly reduced TREM2 levels (Figures 5J–5L). Importantly, this effect was observed exclusively in TCs (Figure 5M), underscoring the necessity of astrocyte-microglial cell communication in modulating TREM2 expression under inflammatory conditions. In accord, we found that APOE protein levels also decreased following treatment of TCs with TNF + IL-1α + C1q (Figure 5N), while SPP1 and GPNMB levels were not significantly changed (Figures S4F and S4G). Collectively, these results indicate that the astrocyte co-culture environment strongly influences proteins linked to DAM.

### fAD neurons induce an inflammatory microglial cell response while dampening astrocyte induction of DAM markers

**Figure 6.**
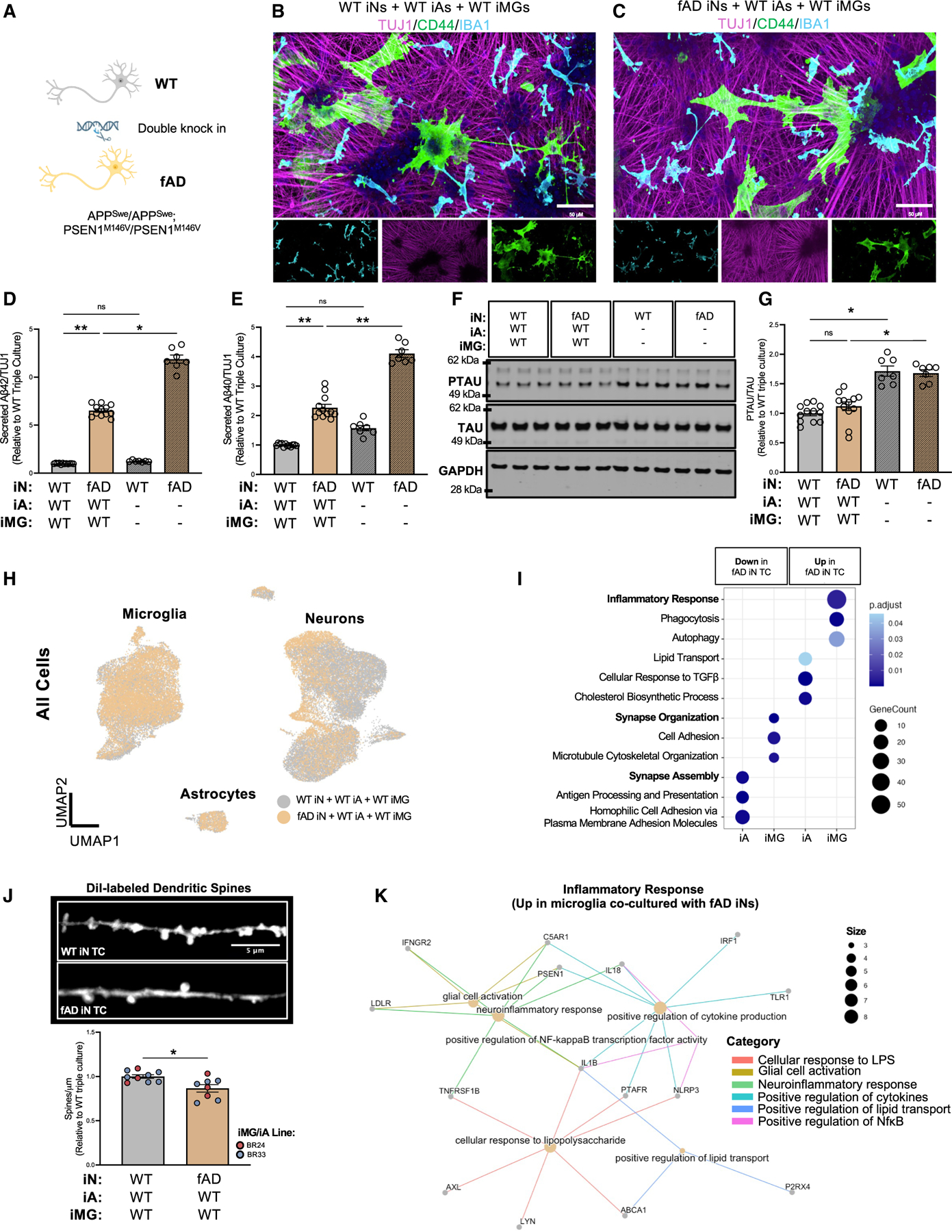
TC with fAD neurons reduces spine density and exacerbates inflammatory signatures in microglia (A–C) fAD or isogenic wild-type (WT) iNs were co-cultured with WT iAs and iMGs. (B and C) Representative immunostaining of iNs (TUJ1, magenta), iMGs (IBA1, cyan), iAs (CD44, green), and nuclei (DAPI, blue). Scale bars, 50 μm. See Figures S5A–S5F for cell-type proportions and additional images across WT and fAD TCs. (D and E) Secreted Aβ42 and Aβ40 (normalized to TUJ1) in WT versus fAD TC and iN MC, relative to WT TC (*N* = 2 genetic backgrounds for glia, 3 differentiations, and 2–4 wells/differentiation). (F and G) Representative WB (one genetic background, technical replicates from a single differentiation) of phosphorylated tau (PTAU^202/205^) and total tau in WT and fAD TCs and MCs, with PTAU:TAU ratios normalized to WT TC (*N* = 2 genetic backgrounds for glia, 3 differentiations, and 2–4 wells/differentiation). (H) scRNA-seq of WT and fAD iNs in TCs with WT iAs/iMGs. UMAP plots of iMGs, iAs, and iNs are colored by WT versus fAD culture identity (one genetic background). See Figures S6A and S6B for labeling by cluster index and fraction of each cluster based upon culture identity. (I) Dot plot showing significantly enriched GO pathways in iAs and iMGs comparing fAD iN TC and WT iN TC within each cell type. Input data are DEGs identified in each cell type, comparing fAD to WT iN TCs. Dots are sized based on the number of DEGs in the indicated pathway and colored based on adjusted *p* value. Complete dataset can be found in Table S6. (J) Dendritic spine density in WT versus fAD iN TCs labeled with DiI. Data are normalized to WT TCs (*N* = 3 differentiations, 2 genetic backgrounds, and 2–3 wells/differentiation), with blinded analysis. Each dot represents the average of five dendrites per well. Scale bar, 5 μm. (K) Gene-concept network of leading-edge genes in the ‘‘inflammatory response’’ pathway, upregulated in microglia co-cultured with fAD iNs (from scRNA-seq in H and I). Data are the mean ± SEM; mixed-effects analysis. ****p < 0.0001, **p < 0.01, *p < 0.05, and ns, not significant.

Next, we evaluated the capacity of our TC model to elicit both known and yet-to-be discovered transcriptional responses in microglia upon exposure to high levels of Aβ by establishing TCs with iNs carrying fAD mutations (Figures 6A–6C and S5A–S5F). In these cultures, ROSMAP astrocytes and microglia that do not carry fAD mutations were used. Compared with isogenic WT iNs, TCs containing fAD iNs exhibited elevated Aβ42 and Aβ40 levels in the media, along with an increased Aβ42:40 ratio (Figures 6D, 6E, and S5G). Notably, extracellular Aβ was significantly lower in TCs than in iN MCs, suggesting enhanced glial uptake and/or degradation (Figures 6D and 6E). Additionally, microglia clustered around X34-labeled Aβ in fAD cultures (Figure S5H). While phosphorylated tau (p202/205; AT8) levels did not differ between fAD and WT TCs, they were reduced in TCs compared to iN MCs (Figures 6F and 6G).

**Figure 7.**
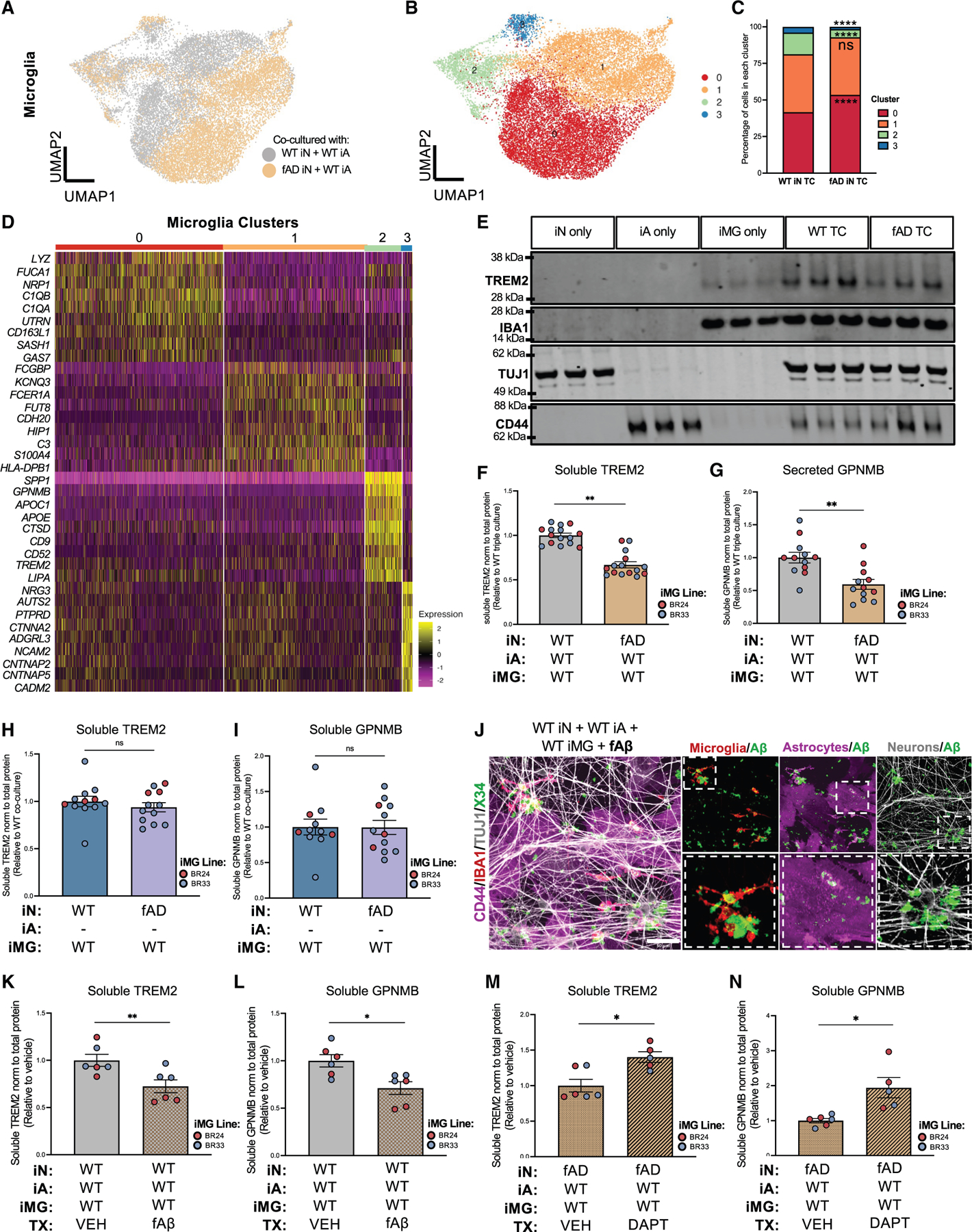
fAD neurons suppress astrocyte-mediated induction of DAM signatures (A and B) UMAP plots of isolated microglial cells from WT and fAD TCs, re-clustered away from neuron/astrocyte expression vectors (from the experiment in Figure 6H). See Figures S6C–S6H for neuron and astrocyte clusters. (C) Proportional composition of each microglial cell subcluster by culture condition (WT versus fAD iNs). Colors match clusters in (B). Chi-squared test, ****p < 0.0001, and ns, not significant. (D) Heatmap of DEGs across the microglial cell subclusters; see Table S6 for the complete dataset. (E) Representative western blot (one genetic background, technical replicates from a single differentiation) of TREM2, IBA1, TUJ1, and CD44 in MC, WT TCs, and fAD TCs. (F–I) Secreted TREM2 and GPNMB (ELISA) in co-culture conditions, normalized to WT within each experiment. *N* = 2 genetic backgrounds, 4–5 differentiations, and 2–3 wells/differentiation. See Figure S7 for APOE and SPP1 data and an additional fAD line. (J) Representative immunofluorescence of WT TCs treated with fibrillar Aβ (fAβ) and labeled for astrocytes (CD44, magenta), neurons (TUJ1, gray), microglia (IBA1, red), and fibrillar Aβ (X34, green). Insets show higher magnification of each cell type’s X34 overlap. Scale bar, 50 μm. (K–N) Secreted TREM2 and GPNMB (ELISA) in TCs treated with fAβ (K and L) or the γ-secretase inhibitor DAPT (M and N). *N* = 2 genetic backgrounds, 2 differentiations, and 2–3 wells/differentiation. See Figure S7 for APOE and SPP1. Data are the mean ± SEM; mixed-effects analysis unless otherwise stated (C uses chi-squared). **p < 0.01, *p < 0.05, and ns, not significant.

We performed scRNA-seq to compare WT and fAD TCs and observed notable shifts in cellular cluster compositions within each major cell type (Figures 6H, 7A–7C, and S6A–S6H; Table S6). To investigate glial responses to high-Aβ-secreting fAD neurons, we performed differential expression analyses within iAs and iMGs co-cultured with either WT or fAD iNs (Table S6). Pathway analyses revealed that synapse organization and synapse assembly pathways were downregulated in both iAs and iMGs co-cultured with fAD neurons (Figure 6I; Table S6). Consistent with findings from AD mouse models showing pronounced dendritic spine loss,^58–60^ we observed a significant reduction in spine density in fAD iN TCs compared to WT TCs (Figure 6J). Moreover, inflammatory response pathways and NF-κB-dependent pathways were upregulated in microglia co-cultured with fAD iNs (Figures 6I and 6K; Table S6). Given that AD mouse models and postmortem human AD brain tissues display upregulated DAM signatures,^4,61,62^ we next investigated whether the DAM-like cluster in iMG TCs was altered in the presence of fAD neurons. UMAP clustering of WT versus fAD iN TCs revealed four major microglial cell subclusters, each associated with distinct functional profiles: phagocytosis and migration (cluster 0: *C1QB*, *C1QA*, and *NRP1*), DAM-like (cluster 2: *APOC1*, *APOE*, *TREM2*, *SPP1*, *GPNMB*, and *CD9*), and synapse-associated (cluster 3: *CNTNAP2*, *CTNNA2*, and *CNTNAP5*) (Figures 7A–7D; Table S6). Surprisingly, the DAM signature was significantly reduced in microglia co-cultured with fAD iNs (Figure 7C). At the protein level, TREM2, APOE, SPP1, and GPNMB also were downregulated (Figures 7E–7G and S7A–S7C), and these changes required the presence of astrocytes (Figures 7H, 7I, S7D, and S7E). Furthermore, treatment of WT TCs with fibrillar Aβ recapitulated some aspects of DAM marker downregulation, while γ-secretase inhibition partially restored expression of TREM2 and GPNMB in fAD TCs (Figures 7J–7N and S7F–S7I). A second fAD neuronal line harboring the APP ‘‘London’’ mutation (V717I) similarly displayed reduced DAM markers compared to its isogenic WT counterpart (Figures S7J–S7N). Collectively, these findings indicate that astrocyte-driven induction of the DAM state is suppressed by Aβ-dependent mechanisms in fAD neurons. This highlights a potential mechanism in which microglial cell-astrocyte crosstalk is critical for the early stages of AD neuropathology.

## DISCUSSION

We present a comprehensive platform for investigating human intercellular interactions, integrating single-cell gene expression and secretome profiling of neurons, astrocytes, and microglia in MCs and TCs. Further, our scRNA-seq dataset provides insights into how fAD neurons influence astrocytes and microglia. Advances in iPSC technology have greatly enhanced disease modeling. However, many existing protocols require over 90 days to develop, posing challenges for large-scale applications. Traditionally, co-cultures involving neurons and astrocytes require an intermediate neural progenitor stage,^26,30^ often necessitating months of culture to reach maturity, and achieving consistent populations is challenging. Our approach utilizes differentiation protocols employing forced expression of NGN2 and SOX9/NFIB transcription factors for neurons and astrocytes, respectively.^31,33^ Importantly, these differentiation protocols incorporate a selection process that minimizes variability across iPSC lines and have been used to consistently generate neurons and astrocytes in a cohort of over 100 genetic backgrounds.^35,47^ A significant improvement in our TC model is the incorporation of cryopreservation at early differentiation stages, enabling the initiation of TCs within just 20 days of thawing each cell type and allowing for quality control assessments prior to the initiation of co-culture (Figure 1F). This advancement facilitates large-scale parallel analyses across different genetic backgrounds and treatment conditions and consistent cell-type ratios.

Here, we investigated an intriguing finding that microglia co-cultured with astrocytes and neurons adopt distinct transcriptional states that mirror the DAM state found in the human brain (Figures 4B–4I). While DAMs are concentrated near Aβ plaques in both individuals with AD^62^ and AD mouse models,^4,61^ whether they are protective or contribute to disease progression is incompletely understood and is thought to vary dynamically across disease stages.^4,63^ A recent study demonstrated a beneficial role of ‘‘neurodegenerative microglia’’ (MGnD) by showing that their induction enhanced plaque clearance in AD mice.^64^ Given that the cellular and molecular events driving AD begin to unfold decades before clinical symptoms (reviewed in Jack et al.^65^), models that capture these early temporal events are essential for biomarker discovery and therapeutic development. Our findings suggest that, in this acute co-culture model, the fAD neuronal environment initially represses the DAM signature, supporting hypotheses that the DAM state may be beneficial early in the disease process.^21,63,64,66–68^ Microglia in TC appear less inflammatory, as indicated by reduced secretion of proinflammatory cytokines in response to LPS (Figure 3D). Additionally, anti-inflammatory cytokines (IL-4 and IL-10) and IFN-γ were upregulated more dramatically in response to LPS in TCs compared to MCs (Figure 3D). Intriguingly, increased IFN-γ has been recently shown to play a beneficial role in inducing MGnD in AD mice.^69^ While some inflammatory processes were upregulated in microglia co-cultured with high-Aβ-secreting neurons, we also observed reduced protein levels of TREM2, APOE, SPP1, and GPNMB (Figures 7A–7G, S7A–S7C, and S7J–S7N), which were partially Aβ dependent (Figures 7J–7N and S7F–S7I). The reduction in TREM2 protein aligns with studies showing that microglia from TREM2-KO mice fail to adopt a DAM signature in response to neuropathological challenges.^20,21^ Our data suggest that early pathogenic interactions, prior to the appearance of plaques, that lead to a downregulation in TREM2 protein levels may significantly alter microglial responses to Aβ and contribute to increased inflammatory processes. The downregulation of DAM upon acute exposure to fAD neurons is especially important since current transgenic mouse models and postmortem human brain studies predominantly represent later stages of AD, and it has been described that the upregulation of the TREM2-dependent DAM stage occurs in later disease stages.^4,57,70,71^

In addition to the diverse transcriptional states observed in our TC system, we discover that astrocytes are essential for initiating a DAM transcriptional program. While recent research has begun to clarify how genetic risk factors and transcriptional regulators influence DAM markers, including TREM2 expression,^11,72,73^ the role of cellular and environmental factors in modulating these expression patterns remains less understood. Notably, we found that treatment with a cytokine cocktail known to induce astrocyte reactivity led to a marked decrease in TREM2 levels only in the presence of astrocytes (Figures 5J–5M), aligning with previous findings that proinflammatory stimuli can suppress TREM2 expression.^56,57^ Astrocyte-derived factors, particularly APOE (which is significantly upregulated in TCs) emerge as promising candidates for inducing the DAM signature. For instance, APOE is a ligand for TREM2,^18^ and its expression is positively associated with TREM2 expression and function in the brain.^19^ Furthermore, similar to TREM2-KO mice, APOE4 mice also lock microglia in a homeostatic state.^64^ Together, these findings highlight the utility of this co-culture model as a versatile platform for dissecting how diverse cellular and genetic contexts shape human microglial, astrocytic, and neuronal states in a cell-type-specific manner.

iMGs are a powerful tool for investigating human disease associated with inflammatory responses and microglial states. Microglia are highly sensitive to their environment, and it is well established that key transcriptional programs are lost when they are removed from the brain and cultured in isolation.^10,74^ Therefore, the upregulation of TREM2 and the DAM-like signature in TC may reflect a physiological level of these genes that diminishes when microglia are cultured in isolation. Recent studies^11^ have demonstrated that exposure to CNS substrates such as synaptosomes and apoptotic cells can induce a DAM-like transcriptional profile in iMGs, even in MC. This suggests that these transcriptional programs are not exclusively linked to Alzheimer’s pathology but may instead represent a broader microglial adaptation that can arise through diverse mechanisms and environmental cues. However, the stability and heterogeneity of these transcriptional changes remain unclear, and additional factors may be required to fully recapitulate a DAM state. While we initially identified this response through single-cell transcriptomics, follow-up analyses at the protein level primarily focused on a subset of DAM-related markers. A more comprehensive approach—integrating broader transcriptomic analyses across varying conditions such as amyloid plaque presence, microglial genetic risk (e.g., variants in *APOE*, *TREM2*, and *INPP5D*), and extended culture durations—will be essential to determine whether distinct or more complete DAM-like microglial states emerge *in vitro*.

### Limitations of the study

The observation of microglial transcriptional states that reflect those in the human brain is compelling and aligns well with recent findings showing that iMGs adopt unique transcriptional responses upon exposure to specific stimuli.^11^ However, it is important to acknowledge that the microglial signatures found in iPSC-derived TCs are not identical to those found in the postmortem human brain, especially homeostatic signatures, which are notoriously difficult to model *in vitro*.^74^ The incorporation of additional cell types and integration of CNS-specific stimuli could significantly enhance our modeling of the diverse transcriptional states present in the human brain. Additionally, the cellular ratios in our model may not perfectly reflect *in vivo* compositions, and the lack of region-specific cellular diversity introduces further limitations. While the use of transcription factor-driven differentiation protocols enables consistency and scalability, they may result in cell identities that are more abstracted than those derived from developmental differentiation methods. Further, future studies incorporating transdifferentiated cells with preserved epigenetic aging signatures could provide additional insights into age-related disease mechanisms. It also is worth noting that the model used here lacks amyloid plaques, which have been shown to strongly influence microglial transcriptional responses *in vivo*. It is therefore possible that the observed transcriptional program represents an early-stage adaptation rather than a fully developed DAM state. Additionally, our study employed a 6-day co-culture period, and studies that extend the co-culture duration will observe how these states evolve over time, providing a fully comprehensive model of disease progression from early to late pathological stages. While the long-term dynamics and plasticity of microglial, neuronal, and astroglial states in co-culture are not yet fully understood, this *in vitro* platform provides a foundation for future longitudinal studies to explore these important questions.

## RESOURCE AVAILABILITY

### Lead contact

Requests for further information and resources should be directed to the lead contact, Tracy Young-Pearse (tpearse@bwh.harvard.edu).

### Materials availability

All unique/stable reagents generated in this study are available from the lead contact with a completed materials transfer agreement (MTA). ROSMAP iPSC lines are available from the New York Stem Cell Foundation through the NYSCF Repository (repository@nyscf.org) with a completed MTA to obtain cohort data and samples (radc.rush.edu).

### Data and code availability

- scRNA-seq data have been deposited in the Gene Expression (GEO) database, with accession number GEO: GSE295330, and proteomics data have been deposited to Synapse, with SynID: syn52977236.
- Expression matrices and meta data for sequencing datasets can be found in the supplemental tables.
- Any additional information required to reanalyze the data reported in this paper is available from the lead contact upon request.

## STAR★METHODS

### EXPERIMENTAL MODEL AND STUDY PARTICIPANT DETAILS

#### Induced pluripotent stem cell lines

IPSC lines were utilized following IRB review and approval through MGB/BWH IRB (#2015P001676). iPSCs were generated from cryopreserved peripheral blood mononuclear cell (PBMC) samples from autopsied participants from the ROS and MAP cohorts. iPSCs were generated using Sendai reprogramming method.^35^ iPSCs undergo a rigorous quality procedure that includes a sterility check, mycoplasma testing, karyotyping, and pluripotency assays performed by the New York Stem Cell Foundation (NYSCF). iPSCs were maintained using StemFlex Medium (Thermo Fisher Scientific). All cell lines were routinely tested for mycoplasma using PCR kit (MP0035–1KT) and STR profiling to prevent potential contamination or alteration to the cell lines. iPSC cell lines harboring two homozygous familial Alzheimer’s disease mutations (APP^SWE^/PSEN1^M146V^;APP^SWE^/PSEN1^M146V^) and its isogenic WT control (Coriell Institute, catalog ID: AG07889) were obtained from NYSCF and are previously described.^36^ An iPSC line bearing the familial Alzheimer’s disease (fAD) London mutation in APP (V717I) and a CRISPR-corrected isogenic control were derived and characterized as described previously.^75^ Briefly, patient fibroblasts carrying the V717I mutation were reprogrammed into iPSCs, and the pathogenic allele was corrected via CRISPR-Cas9 in an isogenic line.

#### Differentiation of iPSCs to induced astrocytes (iAs)

iPSC-derived astrocytes (iAs) were differentiated following a previously published paper^31^ with minor modifications.^35,47,81^ iPSCs were plated at 95k cells/cm^2^ on growth factor reduced Matrigel (Corning #354230) coated plates prior to virus transduction. Then, iPSCs were transduced with three lentiviruses – Tet-O-SOX9-puro (Addgene plasmid #117269), Tet-O-NFIB-hygro (Addgene plasmid #117271), and FUdeltaGW-rtTA (Addgene plasmid #19780). The cells were then replated at 200k cells/cm^2^ using StemFlex Medium (Thermo Fisher Scientific) and ROCK inhibitor (10μM) (D0). The media was changed daily with Expansion Media (EM) from days 1 to 3, and gradually switched from EM to FGF media from day 4 to day 7. Doxycycline (2.5μg/ml, Sigma) was added from day 1 to the end of the differentiation, puromycin (1.25mg/ml, Gibco) was added on days 3 and 4 of the differentiation, and hygromycin (100mg/ml, InvivoGen # ant-hg-1) was added from days 4–6 of the differentiation. On day 8, cells were dissociated using accutase (diluted 1:3 in PBS) and cryopreserved in a 1:1 ratio of 20% DMSO in FBS and FGF media with 10μM ROCK inhibitor and 2.5μg/ml doxycycline. Cryopreserved day 8 stocks were thawed in FGF media with 10μM ROCK inhibitor and 2.5μg/ml doxycycline. On day 9, a full media change in FGF media + 2.5μg/ml doxycycline was performed. Cells were switched to maturation media + 2.5μg/ml doxycycline on day 10 and were fed every 2–3 days in this media until the start of co-culture.

#### Induced astrocyte protocol media

- Expansion Media: DMEM/F12 (Thermo Fisher Scientific), 10% FBS, 1% N2 Supplement (Stemcell Technologies), 1% GlutaMAX (Life Technologies).
- FGF Media: Neurobasal media, 2% B27, 1% NEAA, 1% GlutaMAX, 1% FBS, 8ng/ml FGF, 5ng/ml CNTF, 10ng/ml BMP4.
- Maturation Media: 1:1 DMEM/F12 and neurobasal media, 1%N2, 1%GlutaMAX, 1% Sodium Pyruvate, 5μg/ml N-1% N2, 1%-GlutaMAX, 1%Sodium Pyruvate, 5μg/ml N-acetyl cysteine, 5ng/ml heparin-binding EGF-like GF, 10ng/ml CNTF, 10ng/ml BMP4, 500μg/ml dbcAMP.

#### Differentiation of iPSCs to microglia-like cells (iMGs)

iPSC-derived microglia-like cells (iMGs) were differentiated following a previously published protocol,^32,38^ with minor modifications.^35,39^ iPSCs were plated on growth factor reduced Matrigel (Corning #354230) using StemFlex Medium (Thermo Fisher Scientific) and ROCK inhibitor (10μM). From day 0 to day 12 of differentiation, StemDiff Hematopoietic Kit (Stemcell Technologies) was used to generate hematopoietic precursor cells (HPCs). On day 12, cells were replated at 10k cells/cm^2^ in iMG media supplemented with 3 cytokines (IL-34 (100 ng/mL, Peprotech), TGF-β1 (50 ng/mL, Militenyi Biotech), and M-CSF (25ng/mL, Thermo Fisher Scientific)). From days 12–20, iMG media with freshly added cytokines were added to the culture every other day. On day 20, cells were dissociated with pbs and cryopreserved in BamBanker freezing media (Fisher Scientific). Cryopreserved day 20 iMG stocks were thawed in iMG media + 3 cytokines and plated at 53k cells/cm^2^ in fresh iMG media with 3 cytokines. From day 20 to day 37, iMG media with freshly added 3 cytokines were added to the culture every other day. On day 37, cells are resuspended in iMG media with five cytokines (100ng/mL IL-34, 50ng/mL TGF-β1, 25ng/mL M-CSF, 100 ng/mL, CD200 (Novoprotein) and 100ng/mL CX3CL1 (Peprotech)), supplemented every other day until day 41/start of co-culture.

#### iPSC-derived microglia protocol media

- iMG media: DMEM/F12, 2X insulin-transferrin-selenite, 2X B27, 0.5X N2, 1X GlutaMAX, 1X non-essential amino acids, 400mM monothioglycerol, 5 mg/mL insulin, and 1% Pen-Strep.
- 3 cytokines: 100 ng/mL IL-34 (Peprotech), 50ng/mL TGF-b1 (Miltenyi Biotech), and 25 ng/mL M-CSF (ThermoFisher Scientific).
- 5 cytokines: 100ng/mL IL-34, 50 ng/mL TGF-b1, 25ng/mL M-CSF, 100ng/mL, CD200 (Novoprotein) and 100ng/mL CX3CL1 (Peprotech).

#### Differentiation of iPSCs to induced neurons (iNs)

iPSC-derived neurons (iNs) were differentiated following a previously published paper^33^ with minor modifications.^35,82^ iPSCs were plated at a density of 95k cells/cm^2^ on plates coated with growth factor reduced Matrigel one day prior to virus transduction (Corning #354230). Then, iPSCs were transduced with two lentiviruses – pTet-O-NGN2-puro (Addgene plasmid #52047, a gift from Marius Wernig) and FUdeltaGW-rtTA (Addgene plasmid #19780, a gift from Konrad Hochedlinger). The cells were then replated at 200k cells/cm^2^ using StemFlex Medium (Thermo Fisher Scientific) and ROCK inhibitor (10μM) (day 0). The media was changed to KSR media (day 1), 1:1 of KSR and N2B media (day 2) and N2B media (day 3). Doxycycline (2μg/ml, Sigma) was added from day 1 to the end of the differentiation, and puromycin (5mg/ml, Gibco) was added from day 2 to the end of the differentiation. On day 3, B27 supplement (1:100) (Life Technologies) was added. On day 4, cells were dissociated using accutase (diluted 1:3 in PBS) and cryopreserved in a 1:1 ratio of 20% DMSO in FBS and iN D4 media (NBM media +1:50 B27 + BDNF, GDNF, CNTF (10 ng/mL, Peprotech) with 10μM ROCK inhibitor and 2.0μg/ml doxycycline. One day prior to thawing day 4 stocks, plates were coated overnight at 37°C with poly-L-ornithine (Sigma-Aldrich, #P3655) and laminin (Gibco, #23017015). The next day, the plates were washed with PBS and further coated with Matrigel (Corning, #356234). Cryopreserved day 4 stocks were thawed in iN media with 10μM ROCK inhibitor and 2.0μg/ml doxycycline and plated at 53k cells/cm^2^. From day 4 to the end of differentiation/start of co-culture day 21, cells were cultured in iN media and fed every 3–4 days.

#### Induced neuron protocol media

- KSR media: Knockout DMEM, 15% knockout serum replacement (KOSR), 1x MEM-NEAA, 55 mM beta-mercaptoethanol, 1x GlutaMAX (Life Technologies).
- N2B media: DMEM/F12, 1x GlutaMAX (Life Technologies), 1x N2 supplement B (Stemcell Technologies), 0.3% dextrose (D-(+)-glucose, Sigma).
- NBM media: Neurobasal medium, 0.5x MEM-NEAA, 1x GlutaMAX (Life Technologies), 0.3% dextrose (D-(+)-glucose, Sigma).

#### Optimization of triculture media (TCMs)

iNs, iAs, and iMG monocultures were differentiated as described above. On iN day 22 (or the day of astrocyte replating), the media was switched to iA-iN re-plating media (see below). On iA day 20, iAs were dissociated as described above and re-plated in iA-iN replating media at a density of 53k cells/cm^2^ on plates previously coated with matrigel. On Day 21, iN and iA monocultures were switched to one of the six TCM media compositions (see Table S2). On iMG day 41, iMGs were dissociated as described above and re-plated in one of the six TCM media compositions at a density of 53k cells/cm^2^ on plates coated with matrigel. iN, iA, and iMG monocultures were maintained in TCM for an additional 3 days prior to harvest. As a control, each cell type also had a condition that was maintained in their respective cell type media.

#### iPSC-derived astrocyte-neuron-microglia triculture

To assemble iPSC-derived tricultures, D20 iMGs, D4 iNs, and D8 iAs were thawed according to the timeline outlined in Figure 1F. iMGs, iNs, and iAs were differentiated as described in previous sections. Importantly, D4 iNs were plated in their final destination for triculture experiments on poly-L-ornithine, laminin, and matrigel coated plates as described in the iN differentiation section above. On triculture day 20, iAs were dissociated in accutase (diluted 1:3 in PBS) and re-plated on top of iN monocultures in iA-iN co-culture media at a density of 47K cells/cm^2^. On triculture day 21, iMGs were dissociated in PBS and re-plated on top of iA-iN co-cultures in triculture media at a density of 42K cells/cm^2^. All three cell types were then co-cultured together for up to six additional days, with ½ media changes performed every three days. The exact day of astrocyte and microglia addition (e.g., D20 for iAs and D21 for iMGs) may vary by a few days, provided that each cell type is close to full differentiation at the time of plating into the triculture. The final ratio of cells at the end of co-culture is approximately 6 neurons: 2 astrocytes: 3 microglia (see Figure 1G). Blinded manual quantification of immunostained NeuN (neurons), IBA1 (microglia), and CD44 (astrocytes) nuclei was performed across six fields of view distributed across the well (see Figures 1G and S5C–S5F) using a confocal microscope. Cell numbers were manually counted and categorized based on cell-type marker expression, and ratios were calculated to reflect the relative composition of each cell type in triculture. The final cell-type ratios listed in Figure 1G were derived from three independent co-cultures with two wells per differentiation quantified. Additional qualifications from an independent set of four differentiations are shown in Figures S5A and S5B.

While initial seeding densities approximate a 10:9:8 neuron:astrocyte:microglia ratio, we do not expect these proportions to be maintained throughout the culture period due to:

1. Neuronal Expansion: Neurons are plated at **day 4**, when they are immature and retain some proliferative capacity for the first 1–2 days of culture, leading to a relative increase in neuronal density over time.
2. Astrocyte Attachment Efficiency: Not all iAs adhere post-replating, and unattached cells are removed before microglia addition.
3. Microglial Proliferation: Unlike neurons and astrocytes, microglia retain some proliferative capacity at this differentiation stage, further contributing to shifts in the final cell ratios.

#### iPSC-derived co-culture media

- iA-iN Co-culture/Replating Media: iA maturation media + 1:50 B27 + BDNF, GDNF (10 ng/mL, Peprotech) + Doxycycline (2.5μg/ml, Sigma) + ROCK inhibitor (10 μM).
- Tri-culture Media: BrainPhys Neuronal Medium (StemCell Technology, 5792) + NeuroCult SM1 Neuroonal Supplement (StemCell Technology, 5711) + 100ng/mL IL-34, 50ng/mL TGF-β1, 25ng/mL M-CSF, 100ng/mL CD200 (Novoprotein) and 100ng/mL CX3CL1 (Peprotech).

### METHOD DETAILS

#### Western blotting

Cells were lysed with RIPA lysis buffer (Thermo Fisher Scientific #89900) with the protease inhibitor (Complete TM mini protease inhibitor, Roche) and phosphatase inhibitor (phosphoSTOP, Roche) added freshly before the lysis. Cells were lysed for 30 min on ice before transferring lysates to microcentrifuge tubes. Cell debris was pelleted by centrifugation (13,000 x g) for 10 min at 4°C. Supernatant (cell lysate) was collected and stored at −20°C until use. Protein concentration in cell lysate samples was determined with the Pierce BCA Protein Assay kit (ThermoFisher, #23225). Cell lysates were prepared with 4X LI-COR loading buffer (Fisher Scientific, #NC9779096) and 2.5% β-mercaptoethanol, centrifuged, and incubated at 95°C for 10 min. Samples were resolved using Novex NuPAGE^™^ 4–12% Bis-Tris gels (ThermoFisher, #NP0336BOX) and NuPAGE^™^ 1X MOPS-SDS or MES-SDS running buffer (ThermoFisher, #NP0001). Gel electrophoresis was run at 200V for 50 min. SeeBlue Plus2 (ThermoFisher, #LC5925) pre-stained protein standard was used for evaluation of molecular weight. The gel was extracted and transferred to a nitrocellulose membrane by incubation with 20% methanol tris-glycine transfer buffer at 400mA for 2 h. The transferred blot was blocked with Odyssey blocking buffer (LI-COR, #927–50100) for 1 h at room temperature with agitation and incubated with primary antibody (diluted in blocking buffer) overnight at 4°C with agitation. See Table S7 for dilutions. Blots were incubated with LI-COR secondary antibody diluted 1:10,000 in TBST for 1 h at room temperature with agitation. Blots were washed twice (10 min per wash) with TBST and stored in 1X TBS until imaging. Blots were imaged on a LI-COR Odyssey machine and quantified using ImageStudio software.

#### Immunocytochemistry

Initially, 0.04% PFA was spiked into the wells prior to removing the conditioned media. The plates were then placed at 37°C for 20 min, allowing for gentle initial fixation to avoid microglia loss during subsequent fixation steps. After incubation, cells were washed very gently with PBS and then fixed with 4% paraformaldehyde (PFA, Sigma) for 15 min at room temperature. For experiments involving visualization of amyloid-beta (Aβ), cells underwent X-34 staining (Cell Signaling, Cat #74193). Briefly, a 0.04% X-34 staining solution was prepared by diluting 0.4 mg of X-34 into a mixture of 4 mL ice-cold 200-proof ethanol and 6 mL diH_2_O. After removing PBS, samples were incubated in 200 μL X-34 staining solution for 20 min at room temperature, protected from light. Subsequently, cells were washed with diH_2_O (100 μL) for 3 min at room temperature, followed by a 5-min wash with 0.02 M NaOH solution (50 μL; available from Kellie or prepared as 0.8 g NaOH in 1 L diH_2_O). Cells were then washed twice with PBS (200 μL) for 15–20 min per wash before proceeding. Cells were next blocked in 2% donkey serum (Jackson ImmunoResearch Laboratories) and 0.2% Triton-X-100 (Sigma) in PBS for 1 h at room temperature with gentle agitation. Primary antibodies were diluted in fresh donkey serum blocking buffer, and cells were incubated overnight at 4°C. For dilutions, see Table S7. Following primary incubation, cells were washed three times with PBS and incubated with species-specific secondary antibodies for 1 h at room temperature with agitation. Cells were again washed three times with PBS. Finally, cells were treated with DAPI stain (1:1000 dilution) for 10 min at room temperature with agitation, followed by a final PBS wash in preparation for imaging. Throughout all staining steps, extreme care was taken to minimize agitation force to prevent microglia loss and neuronal peeling. Images were acquired using a Zeiss LSM710 Confocal, Andor Dragonfly 600 Spinning Disk Confocal, or Zeiss LSM880 + Fast Airyscan Confocal microscope.

#### Dendritic spine imaging and analysis

Wells of a 96-well plate fixed with 4% paraformaldehyde underwent dendritic spine labeling using the fluorescent dye 1,1^′^-dioctadecyl-3,3,3^′^,3^′^-tetramethylindocarbocyanine perchlorate (DiI, Invitrogen Cat #D282) as previously described.^83^ Briefly, PBS was removed from each well, and forceps were used to obtain and sprinkle 3–5 Dil crystals across each well. A small amount (∼10μL) of PBS was added to the edge of each well to avoid drying and peeling of cells. Plates were then incubated on an orbital shaker for 10 min at room temperature. Following incubation, wells were washed 2–3 times with PBS, and then wells were left in PBS overnight at 4°C. Approximately 12–18 h later, plates were removed and washed three times (5 min) with diH20 at room temperature. PBS was added to each well, the plates were covered, and incubated at 4°C for at least 72 h prior to imaging to allow for complete dye incorporation into the cells. Images of neuronal dendrites were obtained using the Super Resolution Airyscan function on a Zeiss LSM880 + Fast Airyscan Confocal microscope. Five dendrite images per well were collected using a 63X (oil immersion) objective at 50nm pixel sizes with 0.2μm z-steps, followed by standard Airyscan image processing in Zeiss ZenBlack software. Images were exported as TIFF format (in ImageJ version 2.14.0/1.54f) and imported into NeuronStudio for analysis of dendritic spine densities and morphologies. Spine density measurements were acquired by reconstructing the dendritic cable to acquire the length of each imaged dendrite, followed by manual identification of spines for each dendrite. Mean densities were obtained for each dendrite within a well of a 96-well plate, and final data were analyzed with each well representing an individual data point. Cumulative distributions of assigned spine head diameters (HEAD.DIAMETER) and neck lengths (MAX.DTS) were analyzed by Komogorov-Smirnov tests to evaluate statistical differences of spine morphologies. The data were blinded prior to analysis.

#### Multi-electrode array (MEA)

Cytoview 6-well plates (Axion Biosystems, #M768-tMEA-6B) were coated overnight at 37°C with poly-L-ornithine (Sigma-Aldrich, #P3655) and laminin (Gibco, #23017015). After washing with PBS, wells were further coated with Matrigel (Corning, #356234). Induced neurons (iNs) were plated on day 4 (D4) at a density of 35,000 cells/well, directly on top of the electrodes in 25 μL of iN plating media (see iN protocol above). One hour post-plating, an additional 100 μL of media was added to each well. From D4 to D14, cells were maintained in D4 iN media and fed every 2–3 days. To prevent neuronal detachment, only half-media changes were performed. On D14, a half-media change with BrainPhys Neuronal Medium (StemCell Technologies, #5792) supplemented with NeuroCult SM1 Neuronal Supplement (StemCell Technologies, #5711) was performed, and this medium was used for all subsequent half-media changes until D22. On D22, induced astrocytes (iAs) were added at 30,000 cells/well via a half-media change in BrainPhys + SM1 Supplement with 10 μM ROCK inhibitor (see astrocyte replating details in the previous section). On D23, induced microglia (iMGs) were added at 25,000 cells/well, introduced with a half-media change in BrainPhys + SM1 Supplement containing 200 ng/mL IL-34, 100 ng/mL TGF-β1, 50 ng/mL M-CSF, 200 ng/mL CD200 (Novoprotein), and 200 ng/mL CX3CL1 (Peprotech). Cytokines were added at 2× their standard concentration to compensate for the half-media change. From D26 to D50, cultures were fed every 2–3 days with BrainPhys + SM1 Supplement, maintaining cytokines at normal concentration (100 ng/mL IL-34, 50 ng/mL TGF-β1, 25 ng/mL M-CSF, 100 ng/mL CD200, and 100 ng/mL CX3CL1). Neuronal activity was recorded for 10 min per session on each experimental day. Spikes were detected at 5.5× the standard deviation for each electrode, and hyperactive electrodes were excluded based on a baseline standard deviation >5. Mean firing rate was calculated as the total number of spikes per electrode over the 10-min recording, normalized to the total recording duration. Additional measures of network activity metrics were analyzed using MEA Network Analysis Pipeline (MEA-NAP) as described in Sit et al.^49^ Network-level features such as mean edge weight, node strength, and average controllability were extracted to quantify functional connectivity and network dynamics. Average controllability was used to assess how easily nodes could drive the network into different activity states, while mean edge weight and node strength captured overall connectivity robustness.

#### Synaptic vesicle release assay

Synaptic Vesicle Release was performed as previously described^48^ with minor modifications. Circular pretreated 12mm coverslips (Neuvitro) were coated with Poly-L-Ornithine (used at 20μg/ml, Sigma) and laminin (used at 5μg/ml, Invitrogen) in DPBS (Gibco) as well as matrigel (used at 0.042 mg/cm^2^, Corning) in a 24 well plate (Corning). After coating, coverslips were transferred to ultra-low attachment 24 well plates (Corning). Induced neurons (iNs) were plated from thaw on Day 4 of differentiation onto precoated coverslips at a density of ∼100,000 cells/well. Neurons were maintained with Neural Basal Media (Life Technologies) supplemented with 1:50 B27 (Life Technologies), 10 ng/ml BDNF, 10 ng/ml GDNF, and 10 ng/ml CNTF (Peprotech), 5μg/ml to 10μg/ml puromycin (cell line dependent, Life Technologies), and 2μg/ml doxycycline hyclate (Sigma). On day 15, cells were switched to BrainPhys Neuronal Medium with SM1 supplement (StemCell Technologies). On day 19, cells were transduced with the plasmid SypHy (gift from Pascal Kaeser) packaged into ultra high titer (>10^9^) lentivirus by Alstem. Cells were transduced with 1μL of each lentivirus per well of cells in fresh BrainPhys+SM1 for 24 h. On day 20, the media was completely changed and iAs were re-plated on top of iN cultures at ∼90,000 cells per well as described in the iPSC-derived triculture section above. On day 21, iMGs were re-plated on top of iN-iA co-cultures at ∼80,000 cells per well as described in the iPSC-derived triculture section above. Cells were maintained until Day 27 and then taken for imaging.

To image the cells, coverslips and culture media were transferred to a perfusion chamber (Warner Instruments) with a stage adapter to fit a Zeiss 710 confocal microscope (Warner Instruments). Once on the microscope, the chamber was attached via tubing and manifolds (Warner Instruments) to a syringe stand containing perfusion salt solutions and a bidirectional pump to clear the field (Harvard Apparatus). The cells were perfused with an extracellular solution (140mM NaCl, 5mM KCl, 2mM CaCl2, 2mM MgCl2, 10mM Glucose, 10mM HEPES in MilliQ water) to remove residual media. Synaptic release data was collected over the course of a 140 frame time series in the Zen Black software with the 40x oil objective; from 0–30 s, cells were perfused with extracellular solution; from 30–90 s, cells were perfused with stimulation solution (140 mM NaCl, 140mM KCl, 2mM CaCl2, 2mM MgCl2, 10mM Glucose, 10mM HEPES in MilliQ water); from 90–140 s, cells were perfused with an unquench solution (90mM NaCl, 5mM KCl, 2mM CaCl2, 2mM MgCl2, 10mM glucose, 10mM HEPES, 50mM NH_4_Cl). Following the time series, cells were perfused with extracellular solution once more.

FIJI time series analyzer was used to quantify fluorescence intensity in regions of interest (ROI) over time. ROIs were chosen based upon SypHy fluorescence. The following metrics were calculated for each ROI: F_0_ = mean fluorescence at baseline; F_stim_ = mean fluorescence with stimulation (KCl); F_un_ = max fluorescence at unquench (NH_4_CL); deltaF = F_stim_ minus F_0_. The percent of puncta with a fold chance of F_un_ to F_0_ greater than 2 was calculated, as well as the percent of puncta with a delta F>F_0_, and only those puncta meeting both criteria were included in subsequent analyses. DeltaF to F_un_ was calculated for each timepoint to determine the response to KCL as a percentage of F_un_.

#### ELISAs

72hr conditioned media was collected prior to harvest. Extracellular proteins were measured following manufacturer instructions. Samples were normalized to total protein concentration as determined by the Pierce BCA Protein Assay kit (ThermoFisher, #23225). For TREM2, SPP1, and GPNMB ELISA in triculture, samples were normalized to IBA1. See Table S7 for ELISA kits.

#### Cell culture treatments

For treatments described in Figures 3 and 5, cells were fed with TCM5 media containing the following: 400ng/mL C1q (MyBioSource, MBS147305) + 30 ng/mL TNF (R&D, 210-TA-005) + 3 ng/mL IL1ɑ (R&D, 200-LA-002) for 24 h as previously described^51^ and 100 ng/mL LPS (Sigma, L3012) for 6 h as previously described.^39^ For the 24 h cytokines, the vehicle was 0.1% BSA in PBS and for LPS the vehicle was water. For treatments described in Figure 7, cells were fed with TCM5 media containing 1 μM DAPT (STEMCELL, Cat #NC1730765) or 0.5 μM fibrillar Aβ (preparation described below). Beginning on iN day 18 (3 days prior to addition of astrocytes), DAPT was added to neuron monocultures and was supplemented throughout the culture duration. Fibrillar Aβ was added 1 h after the addition of microglia to the co-culture and was added again for each media change prior to harvest.

#### Aβ fibril preparation

Aβ1–42 monomers (Anaspec) were dissolved in 7 M guanidine hydrochloride overnight at room temperature. The solution was chromatographed on a Superdex 75 Increase column in running buffer 50 mM ammonium bicarbonate pH 8.5, and a single monomer peak was collected, pooled, diluted to 10 μM in TBS (20 M Tris, 500 mM NaCl, pH 7.4) using UV absorbance with extinction coefficient 1,490 M-1 cm-1. To generate fibrils, 0.5 ml SEC-purified monomer was shaken at 800 rpm in a microcentrifuge tube in an Eppendorf tube-shaker at 37°C overnight. The suspension was then centrifuged for 1 h at 100,000 g in a TLA-55 rotor, followed by two washes in cold TBS with the same centrifugation. The final pellet was resuspended to a concentration of 100 μM followed by sonication five times for 5 s at 35% power to disperse. The fibrils were aliquoted and flash-frozen at −80°C.

#### TMT proteomic and data analysis

##### Sample processing

Paired BR24^35^ iMG, iN, and iA monocultures and tricultures were differentiated as described above. The media was removed, spun down at 10,000 g for 10 min, the supernatant was removed and flash frozen on dry ice. Samples were processed as previously described.^39,81^ CM was lysed in 250 μL of urea lysis buffer (8 M urea, 100 mM NaHPO4, pH 8.5) with the protease inhibitor (Complete TM mini protease inhibitor, Roche) and phosphatase inhibitor (phosphoSTOP, Roche) added freshly before the lysis. All homogenization was performed using a Bullet Blender (Next Advance) according to manufacturer protocols. Briefly, each tissue piece was added to urea lysis buffer in a 1.5 mL Rino tube (Next Advance) harboring 750 mg stainless steel beads (0.9–2 mm in diameter) and blended twice for 5 min intervals in the cold room (4 °C). Protein supernatants were transferred to 1.5 mL Eppendorf tubes and sonicated (Sonic Dismembrator, Fisher Scientific) 3 times for 5 s with 15 s intervals of rest at 30% amplitude to disrupt nucleic acids and subsequently vortexed. Protein concentration was determined by the bicinchoninic acid (BCA) method, and samples were frozen in aliquots at –80°C. Protein homogenates (50 μg) treated with 1 mM dithiothreitol (DTT) at 25°C for 30 min, followed by 5 mM iodoacetamide (IAA) at 25°C for 30 min in the dark. Protein mixture was digested overnight with 1:100 (w/w) lysyl endopeptidase (Wako) at room temperature. The samples were then diluted with 50 mM NH4HCO3 to a final concentration of less than 2 M urea and then and further digested overnight with 1:50 (w/w) trypsin (Promega) at 25°C. Resulting peptides were desalted with a Sep-Pak C18 column (Waters) and dried under vacuum.

##### Tandem mass tag (TMT) labeling

Peptides were reconstituted in 100μl of 100mM triethyl ammonium bicarbonate (TEAB) and labeling performed using TMTPro isobaric tags (Thermofisher Scientific, A44520) as previously described.^84,85^ Briefly, the TMT labeling reagents were equilibrated to room temperature, and anhydrous ACN (200mL) was added to each reagent channel. Each channel was gently vortexed for 5min, and then 20mL from each TMT channel was transferred to the peptide solutions and allowed to incubate for 1hr at room temperature. The reaction was quenched with 5%(v/v) hydroxylamine (5 mL) (Pierce). All 16 channels were then combined and dried by SpeedVac (LabConco) to approximately 100mL and diluted with 1 mL of 0.1% (v/v) TFA, then acidified to a final concentration of 1% (v/v) FA and 0.1% (v/v) TFA. Peptides were desalted with a 60 mg HLB plate (Waters). The eluates were then dried to completeness. High pH fractionation was performed essentially as described^86^ with slight modification. Dried samples were re-suspended in high pH loading buffer (0.07% v/v NH4OH, 0.045% v/v FA, 2% v/v ACN) and loaded onto a Water’s BEH (2.1mm 3 150mm with 1.7 mm beads). An Thermo Vanquish UPLC system was used to carry out the fractionation. Solvent A consisted of 0.0175% (v/v) NH_4_OH, 0.01125% (v/v) FA, and 2% (v/v) ACN; solvent B consisted of 0.0175% (v/v) NH_4_OH, 0.01125% (v/v) FA, and 90% (v/v) ACN. The sample elution was performed over a 25 min gradient with a flow rate of 0.6 mL/min with a gradient from 0 to 50% B. A total of 96 individual equal volume fractions were collected across the gradient and dried to completeness using a vacuum centrifugation.

##### Data processing protocol

All raw files were searched using Thermo’s Proteome Discoverer suite (version 2.4.1.15) with Sequest HT. The spectra were searched against a human Uniprot database downloaded August 2020 (86395 target sequences). Search parameters included 10ppm precursor mass window, 0.05 Da product mass window, dynamic modifications methionine (+15.995 Da), deamidated asparagine and glutamine (+0.984 Da), phosphorylated serine, threonine, and tyrosine (+79.966 Da), and static modifications for carbamidomethyl cysteines (+57.021 Da) and N-terminal and Lysine-tagged TMT (+304.207 Da). Percolator was used to filter PSMs to 0.1%. Peptides were grouped using strict parsimony and only razor and unique peptides were used for protein level quantitation. Reporter ions were quantified from MS2 scans using an integration tolerance of 20 ppm with the most confident centroid setting. Only unique and razor (i.e., parsimonious) peptides were considered for quantification.

#### Dissociation for single cell RNA sequencing and library generation and analysis

Cells in MC and TC were derived from the BR33^35^ iPSC strain (89 years old, male, no cognitive impairment) for iMGs, BR24^35^ iPSC strain (90 years old, female, no cognitive impairment) for iAs, and fAD or isogenic WT control^36^ for iNs. All media were removed from all culture conditions (WT iMG + WT iA + WT iN tricultures, WT iMG + WT iA + fAD iN tricultures, and WT iN + WT iA + WT iMG monocultures). For monocultures, cells were differentiated and cultured in parallel to tricultures under the exact same culturing conditions. The WT TC single-cell dataset used here was previously analyzed in comparison to CLU KO TC in a separate study focused on CLU-dependent astrocyte-microglia signaling^34^ and is reanalyzed here in comparison to fAD TC and MC conditions. The samples were processed as previously described with minor modifications.^39^ Briefly, cells were dissociated with a 1:1 mixture of warm trypsin-EDTA:cold StemPro Accutase Cell Dissociation Reagent for 10min at 37°C. Trypsin was quenched with Trypsin Neutralization Solution (ScienCell, Cat# 0113–2) prior to collection of cell suspension. For triculture conditions, four independent culture wells were combined for each culture condition. For monoculture conditions, four independent monocultures for each cell type (iN, iMG, iA) were combined. Cell suspensions were centrifuged for 5 min at 300 x *g* and resuspended in 1 mL of BrainPhys media. The cell pellet was triturated gently with a p1000 tip and passed through a 40μM Flowmi Cell Strainer (Millipore, BAH136800040–50) to remove debris. Cell suspensions were centrifuged at 200 rcf for 5min and resuspended in 0.04% BSA in PBS. Washes were repeated two more times. At the end of the final wash, cell suspension was passed through another 40μM Flowmi Cell Strainer and counted. Each triculture was loaded onto an individual 10X Chromium Chip well (∼64,000 cells per well) and resulting emulsions were used to generate scRNAseq libraries using the Chromium Next GEM Single Cell 3’ v3.1 chemistry. This loading results in a higher percentage of doublets (which are filtered downstream), ultimately yielding ∼20,000 recovered singlet cells per 10x well.

Libraries were multiplexed and sequenced on a NovaSeq to an average depth of >20K reads per cell based on estimated yields. Fastq files were mapped using the 10xGenomics Cellranger (v7.0.0) pipeline and a GRCh38 index and resulted in an average of >4K mapped and filtered reads per cell. Mappings and counts were analyzed using the Seurat package (v5.0.3) in R (v4.4.1) using RStudio (v1.4.1103). Briefly, imported Cellranger data were filtered to remove cells with <1000 or > 5000 mapped genes and with > 15% mapping to mitochondrial genes, and to remove suspected doublets. The remaining dataset has 58,322 cells (25,150 iMGs, 32,104 iNs, and 1078 iAs) with an average number of mapped genes detected per cell of 2,104 for iMGs, 1,380 for iNs, and 2,748 for iAs, and average total unique molecular identifiers (UMIs) per cell of 4,254 iMGs, 4,402 iNs, and 5,882 iAs. After filtering, normalized and scaled data were clustered using the 2000 most highly variable features. Uniform Manifold Approximation and Projection (UMAP) was run on the first 20 principal components. DEGs between cell groups were identified using FindMarkers function in Seurat using the Wilcoxon Rank Sum test with multiple comparisons adjusted (FDR) *p* value cutoff of 0.05.

#### Gene set enrichment analysis (GSEA)

Gene set enrichment analysis (queried against the Broad Hallmark gene set) was performed using the R package fgsea^87^ and results were plotted using the R package ggplot2.

#### Data visualization

Schematics were generated using Biorender. Graphs and heat maps were generated using R Studio^76^ or GraphPad Prism 10.

### QUANTIFICATION AND STATISTICAL ANALYSIS

Information regarding number of differentiations, lines used, and statistical analyses can be found in the figure legends and Table S1. All statistical tests were performed using GraphPad Prism 10 or R Studio. Statistical analysis for cell culture experiments across multiple differentiations were performed using a two-way mixed-effects analysis in prism with multiple comparisons. Data were arranged in a grouped format, with culture type (e.g., MC vs TC) as the between-subject factor (columns) and differentiation (e.g., differentiations 1 – 4) as the within-subject factor (rows). A mixed-effects model was selected under ‘‘Repeated Measures,’’ and a full model was fit (column/culture type effect, row/differentiation effect, and column/culture x row/differentiation interaction). The asterisks presented in bar graphs represent the statistical significance of the column/culture type effect. For experiments comparing only two groups (MC vs TC), no formal multiple-comparison procedure was performed beyond the main effect. For experiments involving more than two groups, post hoc comparisons were made using Prism’s built-in Tukey’s test, comparing each column mean to every other column mean. Data are plotted as mean ± SEM unless otherwise indicated.

## Supplementary Material

1

2

3

4

5

6

7

8

SUPPLEMENTAL INFORMATION

Supplemental information can be found online at https://doi.org/10.1016/j.celrep.2025.115777.

## Figures and Tables

**Table:** KEY RESOURCES TABLE

REAGENT or RESOURCE	SOURCE	IDENTIFIER
Antibodies		
Mouse anti-GAPDH	Proteintech	Cat# 60004–1-Ig; RRID: AB_2107436
Chicken anti-GFAP	Abcam	Cat# ab4674; RRID: AB_304558
Goat anti-AIF1	Abcam	Cat# ab5076; RRID: AB_2224402
Mouse anti-IkBa	Cell Signaling Technologies	Cat# 4814S; RRID: AB_390781
Rabbit anti-S100B	Abcam	Cat# ab52642; RRID: AB_882426
Chicken anti-TUJ1	Novus	Cat# nb100–1612; RRID: AB_10000548
Rabbit anti-TAU	Cell Signaling Technologies	Cat# 46687S; RRID:AB_2783844
Rabbit anti-TREM2	Abcam	Cat# ab209814; RRID: AB_3095849
Mouse anti-vimentin	Millipore	Cat# CBL202; RRID: AB_93387
Mouse anti-CD44	Abcam	Cat# ab254530; RRID: AB_2885131
Rabbit anti-INPP5D	Cell Signaling Technologies	Cat# 2727S; RRID: AB_2126136
Mouse anti-Cd11b	ProteinTech	Cat# 66519–1; RRID: AB_2881882
Rabbit anti-NeuN	Abcam	Cat # ab177487; RRID: AB_2532109
X-34	Cell Signaling	Cat #74193
Bacterial and virus strains		
pTet-O-NGN2-puro	Zhang et al.^33^	Addgene plasmid #52047
FUdeltaGW-rtTA	Zhang et al.^33^	Addgene plasmid #19780
Tet-O-SOX9-puro	Canals et al.^31^	Addgene plasmid #117269
Tet-O-NFIB-hygro	Canals et al.^31^	Addgene plasmid #117271
SypHy	Wu et al.^48^	Addgene plasmid #82540
Chemicals, peptides, and recombinant proteins		
TNF	Fisher Scientific	Cat #210TA020
LPS	Sigma	Cat #L3012
IL1a	R&D	Cat# 200-LA-002
C1q	MyBioSource	Cat# MBS147305
1,1^′^ -dioctadecyl-3,3,3^′^,3^′^ - tetramethylindocarbocyanine perchlorate	Invitrogen	Cat# D282
DAPT	STEMCELL Technologies	Cat #NC1730765
Fibrillar Aβ	This study	N/A
Critical commercial assays		
R-PLEX Human ApoE Assay	Meso Scale Discovery	cat. #K1512IR-2
R-PLEX Human SPP1 Assay	Meso Scale Discovery	cat. #K151YMR-2
R-PLEX Human GPNMB Assay	Meso Scale Discovery	cat. #K151ZHR
V-PLEX Human Proinflammatory Panel 1	Meso Scale Discovery	cat. #15049D
TREM2 ELISA	Abcam	cat. #ab224881
Deposited data		
Mass spectrometry proteomics data	This paper	Synapse: Syn52977236
scRNAseq data	This paper	GEO: GSE295330
Experimental models: Cell lines		
HUMAN IPSC LINE: BR24, 93% European ancestry	Lagomarsino et al.^35^	BR24, AJ0040
HUMAN IPSC LINE: BR33, 91% European ancestry	Lagomarsino et al.^35^	BR33, AJ0047
7889-SA (WT)	Paquet et al.^36^	N/A
7889-SA (APP^SWE^; PSEN1^M146V^)	Paquet et al.^36^	N/A
APP V717I	Muratore et al.^75^	N/A
APP V717I corrected to WT	Muratore et al.^75^	N/A
Software and algorithms		
V4.4.0 of R	R Core Team^76^	https://www.rstudio.com
CellRanger, v7.1.0	Zheng et al.^77^	https://www.10xgenomics.com/support/software/cell-ranger/latest
Seurat, v5.0.3	Satija et al.^78^	https://satijalab.org/seurat/
Fiji (ImageJ), v2.14.0/1.54f	Schindelin et al.^79^	https://imagej.net/software/fiji/
Prism, v10	Graphpad Software	https://www.graphpad.com
NeuronStudio, v0.9.92	Rodriguez et al.^80^	https://www.bionity.com/en/encyclopedia/Neuronstudio.html
Image Studio Lite, v4.0	Image Studio Software	https://www.licor.com/bio/image-studio/
Biorender	N/A	https://www.biorender.com
Cell Profiler, v4.1.1	Sterling et al.^36^	https://cellprofiler.org/

## Highlights

- Development of a robust, reproducible iPSC-derived human triple-culture platform
- Co-culture enhances transcriptional diversity and functional specialization
- Astrocytes induce human disease-associated microglial states
- Acute exposure to fAD neurons decreases TREM2, GPNMB, APOE, and SPP1 protein levels
