# Supplementary material for: Astrocyte induction of disease-associated microglia is suppressed by acute exposure to fAD neurons in human iPSC triple cultures
Source: Cell Rep. Author manuscript; Available in PMC 2025 Jul 22. (PMC12282607; doi:10.1016/j.celrep.2025.115777)
Supplement: 1 [file NIHMS2092653-supplement-1.pdf]

Cell Reports, Volume *44*

Supplemental information

Astrocyte induction of disease-associated microglia is suppressed by acute exposure to fAD neurons in human iPSC triple cultures

Alexandra M. Lish, Nancy Ashour, Richard V. Pearse II, Paige C. Galle, Gwendolyn A. Orme, Sarah E. Heuer, Courtney R. Benoit, Kellianne D. Alexander, Elyssa F.L. Grogan, Gizem Terzioglu, Allegra Scarpa, Andrew M. Stern, Nicholas Seyfried, Vilas Menon, and Tracy L. Young-Pearse

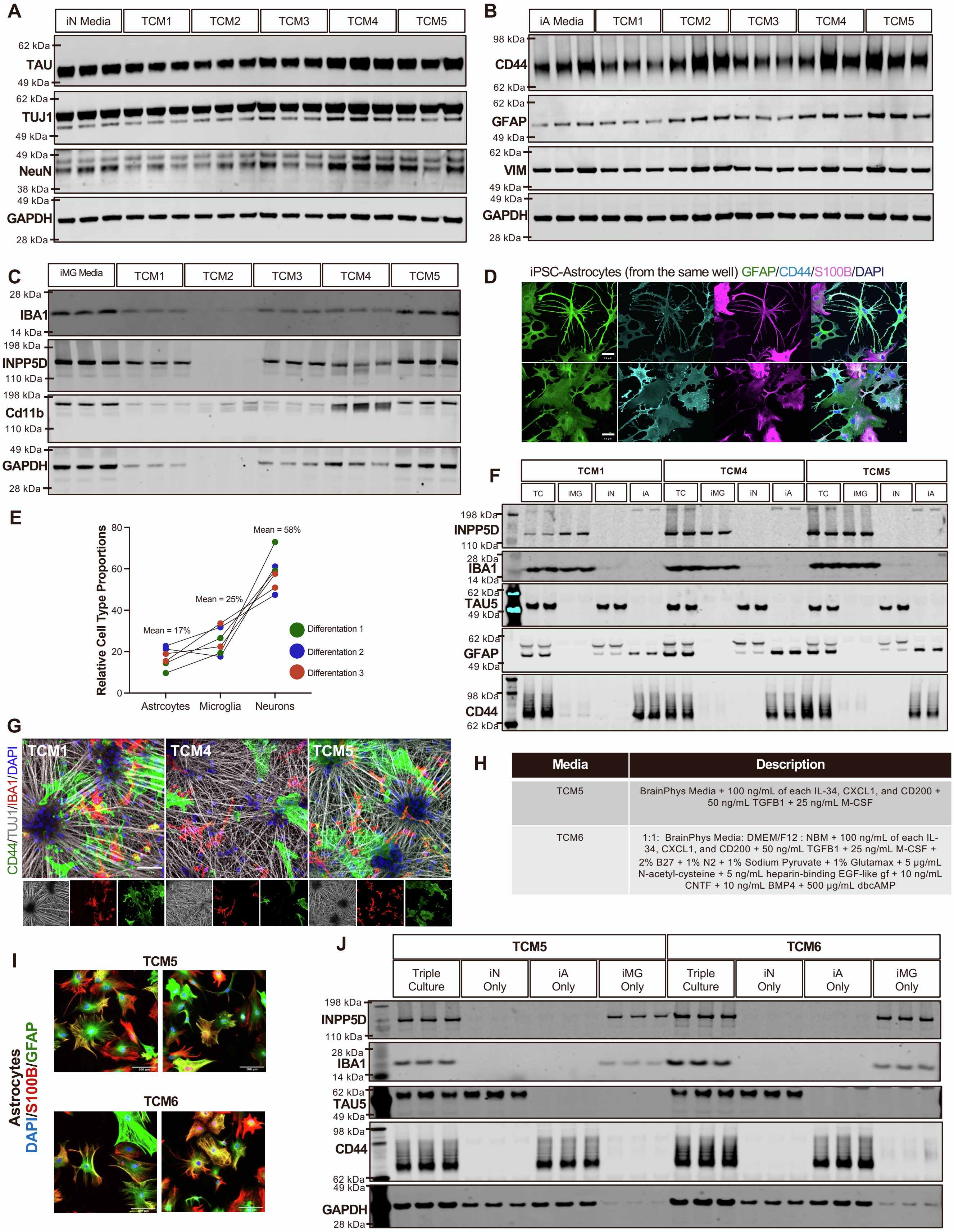

Media	Description
TCM5	BrainPhys Media + 100 ng/mL of each IL-34, CXCL1, and CD200 + 50 ng/mL TGFB1 + 25 ng/mL M-CSF
TCM6	1:1: BrainPhys Media: DMEM/F12 : NBM + 100 ng/mL of each IL-34, CXCL1, and CD200 + 50 ng/mL TGFB1 + 25 ng/mL M-CSF + 2% B27 + 1% N2 + 1% Sodium Pyruvate + 1% Glutamax + 5 µg/mL N-acetyl-cysteine + 5 ng/mL heparin-binding EGF-like gf + 10 ng/mL CNTF + 10 ng/mL BMP4 + 500 µg/mL dbcAMP

Figure S1: TCM optimization for co-culture of astrocytes, neurons, and microglia, related to Figure 1. (A) Representative Western blot (WB) of neurons in iN media and TCM conditions, probed for TAU, TUJ1, NeuN, and GAPDH. (B) Representative WB of astrocytes in iA media and TCM conditions probed for CD44, GFAP, VIM, and GAPDH. (C) Representative WB of microglia in iMG media and TCM conditions probed for IBA1, INPP5D, CD11b, and GAPDH. (D) Representative immunostaining images of iAs in maturation media, highlighting the heterogenous morphologies identified within the same well. Stains represent GFAP (green), CD44 (cyan), S100B (magenta), and DAPI (blue). Scale bar = 50 µM. (E) Individual well–level cell counts corresponding to Figure 1G, color-coded by differentiation batch. Each dot represents a single well, and lines connect each cell type from the same well. (F) Representative WB of triple cultures (TC), microglia monocultures (iMG), neuron monocultures (iN), and astrocyte monocultures (iA) across three TCM conditions, probed for microglial markers (INPP5D, IBA1), neuronal marker (MAPT/TAU), astrocyte markers (GFAP, CD44), and GAPDH. (G) Representative images of triple cultures in three TCM conditions, immunostained for CD44 (astrocytes), IBA1 (microglia), and TUJ1 (neurons). Scale bar = 100 µM. (H) Table describing TCM5 and TCM6 composition. See Supplementary Table S2 for full descriptions. (I) Immunostaining of astrocytes cultured in TCM5 and TCM6, stained for S100B (red), GFAP (green), and DAPI (blue). Scale bar = 1000 µM. (J) Representative WB of INPP5D, IBA1, TAU5, CD44, and GAPDH in MCs and TCs cultured in TCM5 and TCM6.

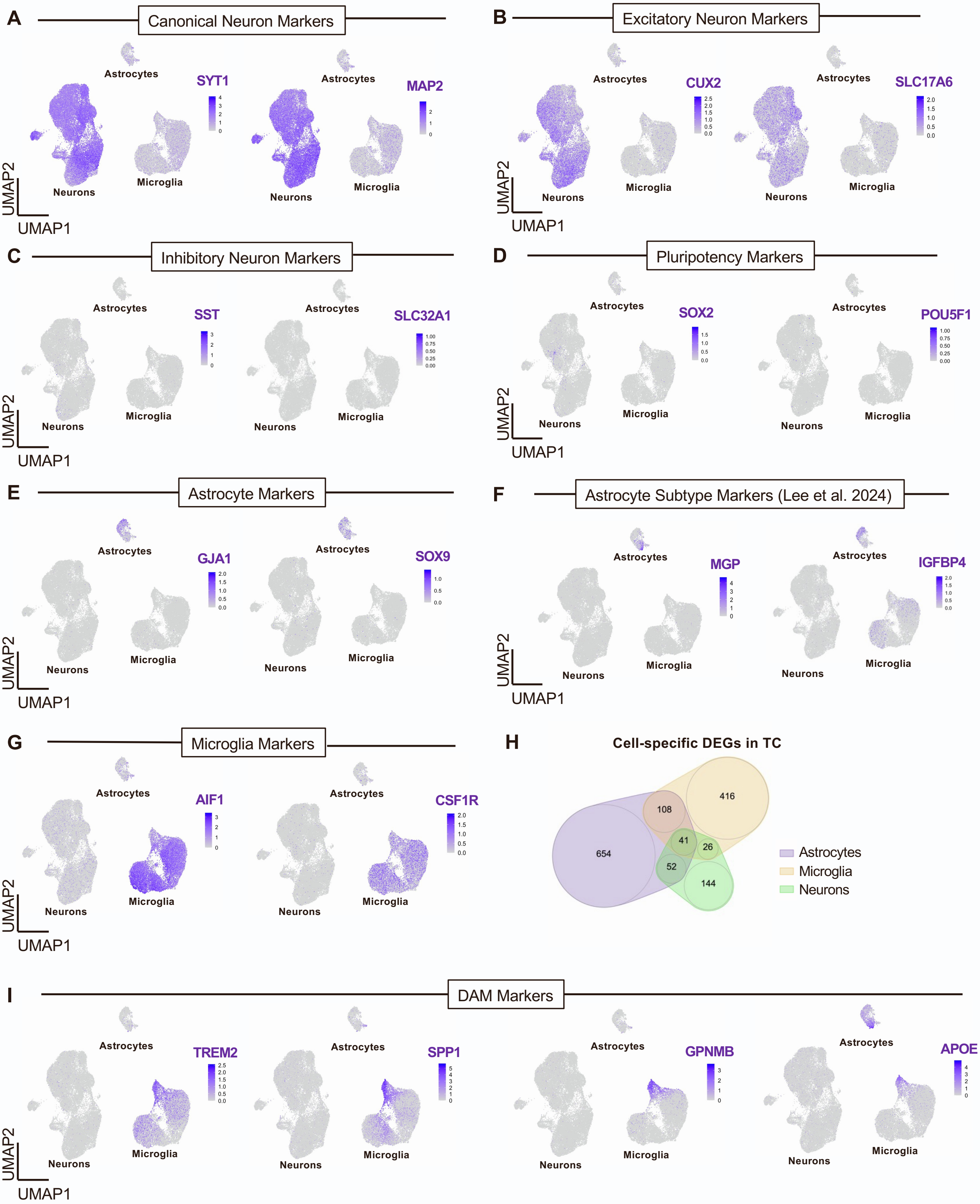

Figure S2: Cell type markers in single-cell RNAseq of iNs, iAs, and iMGs, related to Figure 2 . (A–G, I) UMAP feature expression plots for the scRNAseq dataset of iNs, iAs, and iMGs in MC and TC. Shown are canonical neuron markers (A), excitatory neuron markers (B), inhibitory neuron markers (C), pluripotency markers (D), astrocyte markers (E), iPSC-astrocyte subtype markers previously identified by our group[46] (F), microglia markers (G), and DAM markers (I). Cells are from paired MC and TCs. (H) Venn diagram showing the number of differentially expressed genes (DEGs) in each cell type comparing MC vs. TC (adjusted $p < 0.05$, $|\mathrm{logFC}| > 0.25$, expressed in ≥10% of cells). Data is relevant to scRNAseq experiment depicted in Figures 2A–F. See Tables S3 for complete datasets.

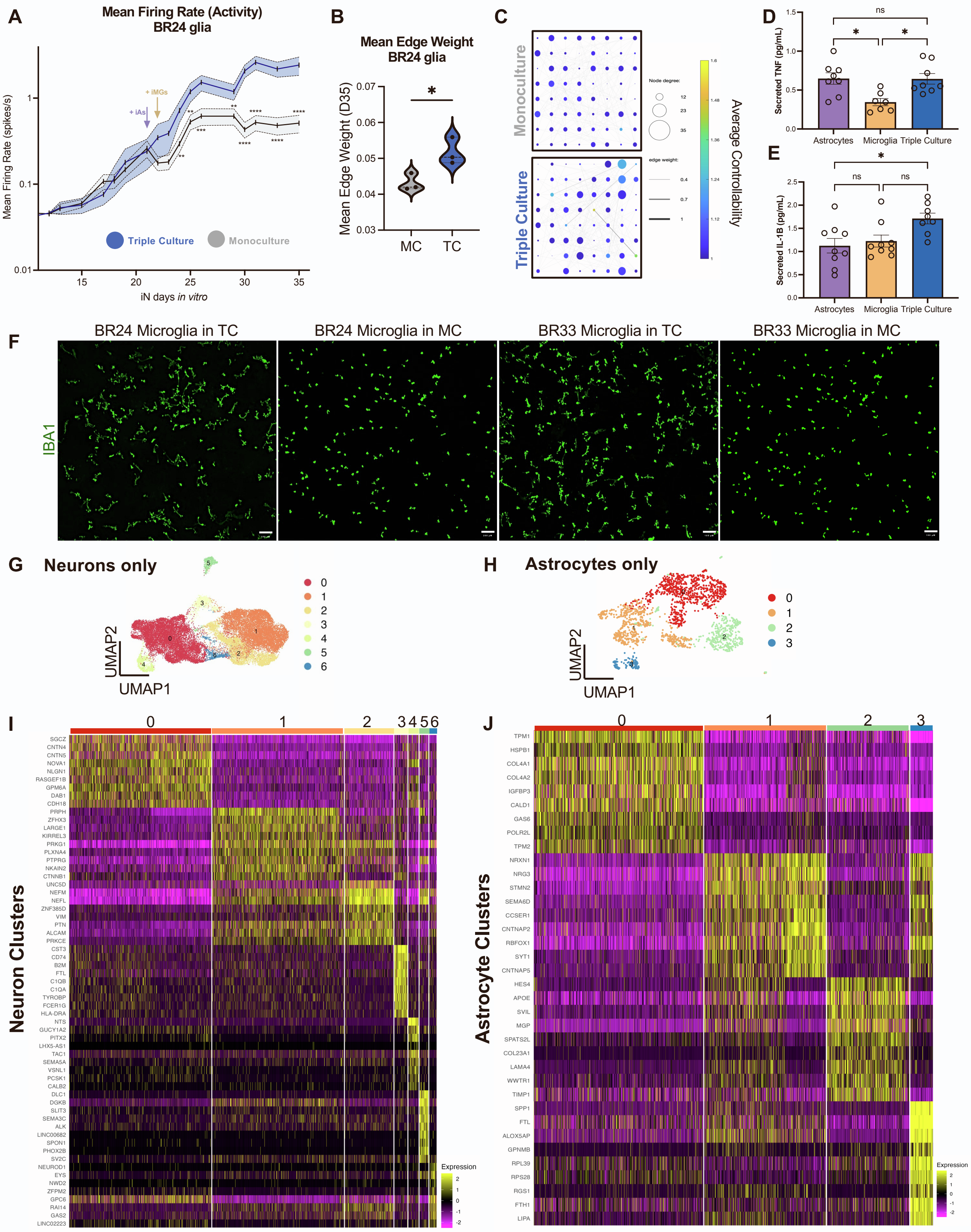

Figure S3: TC-induced changes in neuronal activity and scRNAseq cluster identity for neurons and astrocytes, related to Figure 2. (A) Mean firing rate (spikes/s) of neurons from day 11 to day 35 in MC vs. TC, recorded on Axion multi-electrode arrays (MEAs) (64 electrodes/well, 4 wells/condition, 1 differentiation). BR24 glia cells were used. Mixed-effects analysis with Sidak's multiple comparisons, ****p<0.0001, ***p<0.001, **p<0.01, and *p<0.05. (B) Mean edge weight (a measure of connectivity strength) on iN Day 35 comparing TC to MC. BR24 glia cells were used. N = 64 electrodes per well, 4 wells per condition. Dots correspond to each individual well. Unpaired student's t-test, *p<0.05. (C) Representative network graphs on iN Day 35 illustrating microscale network organization in MC and TC. Node strength (circle size) represents the influence of individual electrodes, edge weight (line thickness) indicates connection strength, and node color reflects average controllability, which quantifies the ability of individual nodes to facilitate transitions between different network states. (D–E) Secreted levels of TNF and IL1β in paired MC and TCs were measured via ELISA. Data presented here is the same as depicted in Figure 3B, except MC values are broken down by iA MC and iMG MC. N = 2 genetic backgrounds, 3 differentiations per genetic background, 2–3 independent wells per differentiation. (F) Representative immunostaining (IBA1, green) of microglia in MC and TC from two genetic backgrounds. Scale bar = 200 μM. (G–H) UMAP plot of neuron and astrocyte cells labeled by subcluster identity. ScRNAseq data in this figure is from the experiment depicted in Figure 2. Colors correspond to cluster indexes. (I–J) Heatmap of DEGs for each cluster, organized by similarity and neuron (I) or astrocyte (J) states.

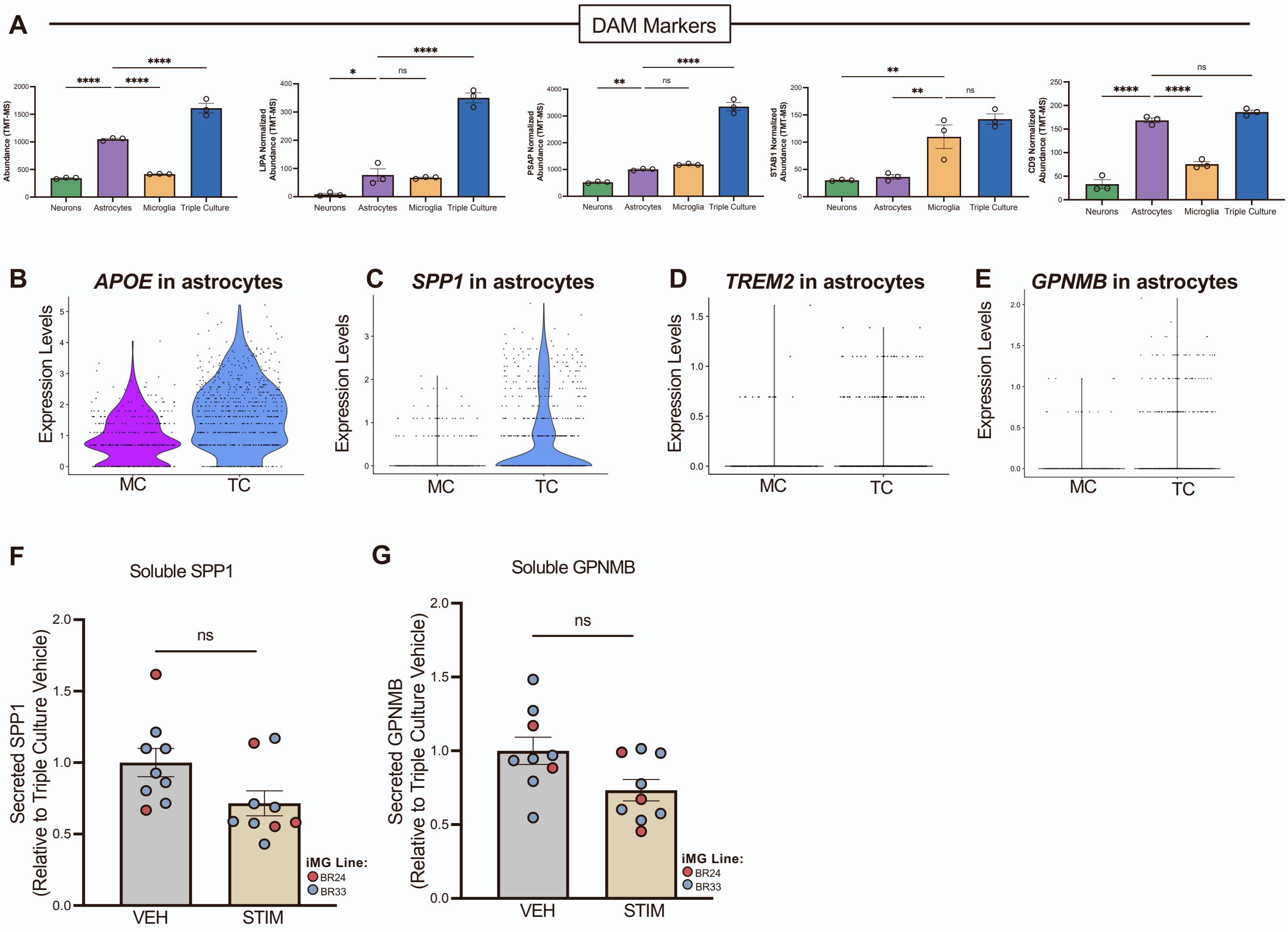

Figure S4. Additional characterization of DAM markers in the tri-culture environment, related to Figure 4. (A) Expression levels of DAM markers identified in microglia cluster 3 (Figure 4C), measured by TMT-MS secretome profiling (data from Figure 3F). One-way ANOVA with Sidak's multiple comparisons. (B–E) Violin plots of APOE, SPP1, TREM2, and GPNMB transcripts in astrocytes under MC vs. TC conditions, derived from the scRNA-seq experiment in Figure 2. (F–G) Soluble SPP1 and GPNMB (ELISA) in iMG MC and TCs treated with vehicle or TNF + IL-1α + C1q (STIM). N = 2 genetic backgrounds, 3 differentiations, 2–3 wells/differentiation. Data is related to experiments detailed in Figure 5J–N.

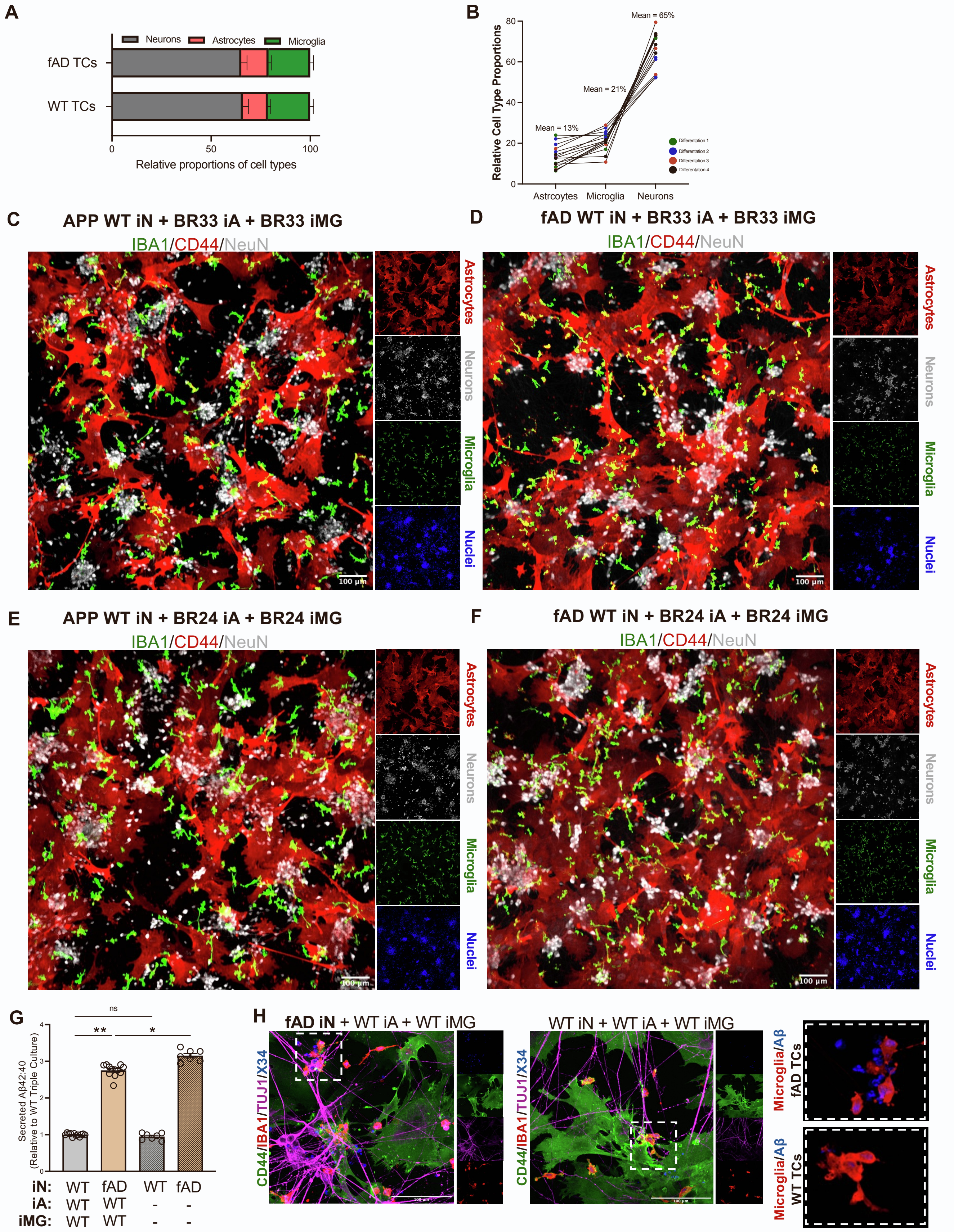

Figure S5. Cell type proportions are unchanged in WT and fAD TCs, related to Figure 6. (A) Bar plot showing the relative percentages of NeuN+ (neurons), IBA1+ (microglia), and CD44+ (astrocytes) cells at day 27, determined from immunostaining (n=4 differentiations, 4 wells per differentiation). Error bars represent standard error. Representative fields of views (FOV) are shown in C–F, with six FOVs per well analyzed by blinded quantification. (B) Individual well–level cell counts corresponding to (A), color-coded by differentiation batch. Each dot represents a single well, and lines connect each cell type from the same well. (C–F) Representative immunostaining images across WT and fAD iN TCs with two different genetic backgrounds for glial cells. Astrocytes are shown in red (CD44), microglia in green (IBA1), and neurons in gray (NeuN). Scale bar = 200 μM. (G) Secreted Aβ42:40 in WT vs. fAD TC and iN MC, relative to WT TC (N = 2 genetic backgrounds for glia, 3 differentiations, 2–3 wells/differentiation). (H) Representative immunofluorescence of WT and fAD TCs labeled for astrocytes (CD44, green), neurons (TUJ1, magenta), microglia (IBA1, red), and X34 (blue). Insets show higher magnification of IBA1 and X34. Scale bar = 100 μM.

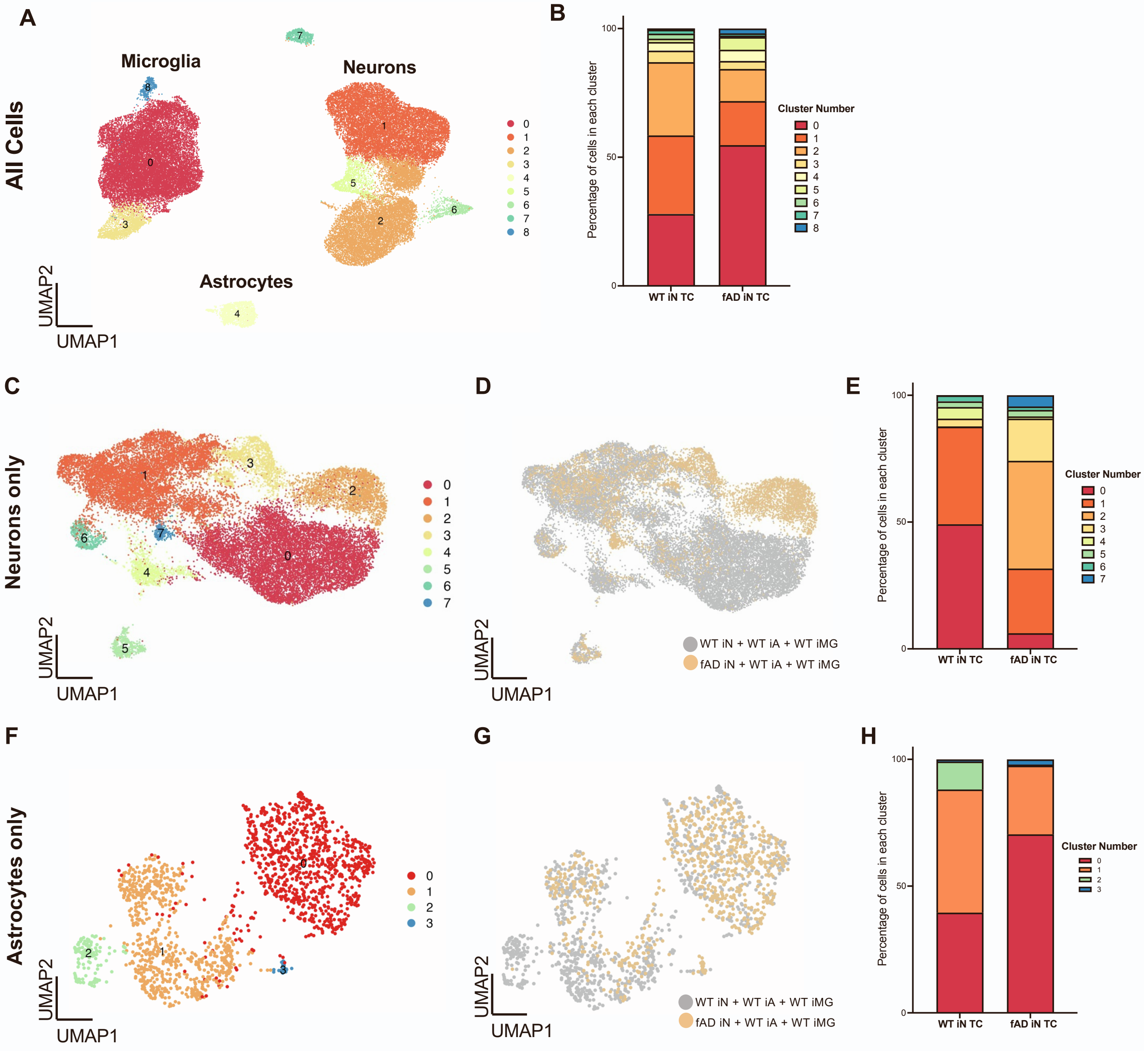

Figure S6. Characterization of scRNAseq data of tri-cultures with fAD and WT neurons, related to Figure 6. (A–B) UMAP plot of iMGs, iAs, and iNs generated from scRNAseq of WT and fAD triple cultures. ScRNAseq data in this figure is from the experiment depicted in Figure 6. Clusters are defined by marker expression and cells are colored by cluster index. Fractions of each cluster based upon culture identity is shown in B. (C–H) UMAP plots of neuron and astrocyte clusters generated from scRNAseq of WT and fAD iN TCs. Cell types were isolated from neuron and astrocyte identities portrayed in Figure 6H/S6A. Clusters are defined by marker expression and cells are colored by cluster index (C,F) or by culture composition (D,G). Fractions of each cluster based upon culture identity are shown in E and H.

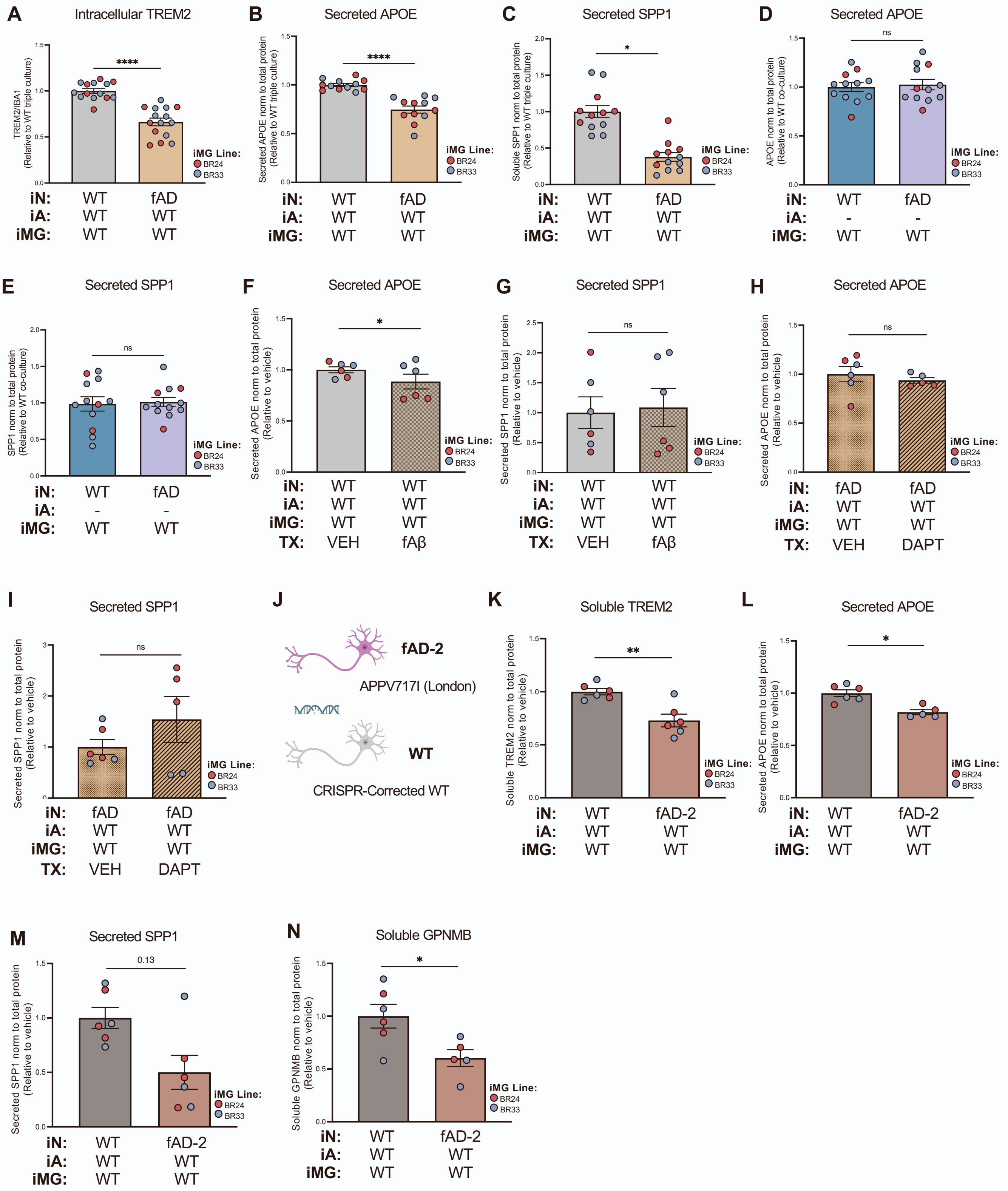

Figure S7. Additional characterization of DAM across experimental conditions, related to Figure 7. (A–E) Quantification of intracellular TREM2 (WB) and secreted (ELISA) SPP1 and APOE levels across co-culture conditions. Data are normalized to WT cultures within each experiment. N = 2 genetic backgrounds, 4–5 differentiations, 2–3 independent wells per differentiation. (F–I) Secreted APOE and SPP1 (ELISA) in co-cultures treated with fAβ (F–G) or the γ-secretase inhibitor DAPT (H–I). N = 2 genetic backgrounds, 2 differentiations, 2–3 wells/differentiation. (J) Schematic overview of additional lines used to interrogate markers associated with DAM. fAD-2 cell line is from a patient harboring the London fAD amyloid precursor protein mutation. CRISPR/Cas9 was used to generate a corrected isogenic WT control. The generation of these lines are previously described.[73] (K–N) Secreted TREM2, APOE, SPP1, and GPNMB (ELISA) across WT and fAD-2 TCs, values are relative to WT cultures. N = 2 genetic backgrounds, 2 differentiations, 2–3 wells/differentiation. Data presented as mean values ± SEM. Mixed-effect model analysis, ****p<0.0001, **p<0.01, *p<0.05, and ns = not significant.
